# The prevalence of diabetes and thyroid related autoantibodies in Sri Lankan children with type 1 diabetes and their unaffected siblings – The utility of a new screening assay

**DOI:** 10.3389/fendo.2023.1028285

**Published:** 2023-02-06

**Authors:** Navoda Atapattu, Marie Amoroso, Michael Powell, D. G. Harendra de Silva, K. Shamya H. de Silva, Jadwiga Furmaniak, Bernard Rees Smith, Lakdasa D. Premawardhana

**Affiliations:** ^1^ Endocrinology and Diabetes Unit, Lady Ridgeway Hospital, Colombo, Sri Lanka; ^2^ FIRS Laboratories, RSR Ltd., Cardiff, United Kingdom; ^3^ Department of Paediatrics, Lady Ridgeway Hospital and Faculty of Medicine, Colombo, Sri Lanka; ^4^ Thyroid Research Group, Cardiff University School of Medicine, Cardiff, United Kingdom

**Keywords:** type 1 diabetes, GADA, IA-2A, ZnT8A, thyroid autoantibodies

## Abstract

**Background:**

There is limited information about diabetes and thyroid related autoantibodies in children with type 1 diabetes (T1D) or their siblings in Sri Lanka.

**Objectives:**

To assess in T1D children and their unaffected siblings the prevalence of autoantibodies to (1) glutamic acid decarboxylase (GADA), insulinoma associated antigen-2 (IA-2A) and zinc transporter 8 (ZnT8A) using 3 Screen ICA™ (3-Screen) and individual ELISA assays; (2) insulin (IAA); and (3) thyroid peroxidase (TPOA), thyroglobulin (TgA) and the TSH receptor (TSHRA).

**Methods:**

We selected - (a) consecutive T1D children, and (b) their unaffected siblings of both sexes, from the T1D Registry at Lady Ridgeway Hospital, Colombo.

**Results:**

The median age (IQR) of 235 T1D children and 252 unaffected siblings was 11 (8.4, 13.2) and 9 (5.4, 14.9) years respectively, and the duration of T1D was 23 (7, 54) months. (1) T1D children (a) 79.1% were 3-Screen positive; (b) all 3-Screen positives were individual antibody positive (GADA in 74%; IA-2A 31.1%; ZnT8A 38.7%); (c) and were younger (p=0.01 vs 3-Screen negatives); (d) multiple autoantibodies were present in 45.1%; (e) IA-2A (p=0.002) and ZnT8A (p=0.006) prevalence decreased with T1D duration. (f) TPOA and TgA prevalence was higher in T1D children compared to unaffected siblings (28%, p=0.001 and 31%, p=0.004, respectively). (2) Unaffected siblings (a) 6.3% were 3-Screen positive (p=0.001 vs T1D), and 2.4% were positive for IAA; (b) four subjects had two diabetes related autoantibodies, one of whom developed dysglycaemia during follow-up.

**Conclusions:**

The 3-Screen assay, used for the first time in Sri Lankan T1D children and their siblings as a screening tool, shows a high prevalence of T1D related Abs with a high correlation with individual assays, and is also a helpful tool in screening unaffected siblings for future T1D risk. The higher prevalence of thyroid autoantibodies in T1D children is consistent with polyglandular autoimmunity.

## Introduction

### Background

Childhood type 1 diabetes mellitus (T1D) is an autoimmune disease with multiple factors affecting its pathophysiology and onset (1). This autoimmune process is precipitated by environmental factors, causes destruction of pancreatic beta cells in genetically predisposed individuals and results in dysglycaemia (2). However, the role of autoimmunity in this process is only partially understood, and genetic predetermination is exemplified by subjects with HLA phenotypes at higher risk for developing T1D (3). T1D incidence is increasing worldwide (3% annually), mostly in children under 5 years of age, potentially leading to a doubling of its global incidence in the next decade (4). Its incidence varies significantly amongst populations in T1D registries (4, 5). The incidence of T1D in Sri Lanka is unknown although evidence suggests it is increasing (6).

Autoantibodies to islet cell antigens are a hallmark of T1D and are directed against the 65kDa isoform of glutamic acid decarboxylase (GADA), insulin (IAA), insulinoma associated antigen-2 (IA-2A) and zinc transporter 8 (ZnT8A) (7, 8). These autoantibodies predate the onset of clinical T1D, are detectable in the blood during infancy and persist for many years thereafter (1, 7). Their clinical utility is therefore high in –

(a) diagnosing T1D - there are only sparse data about T1D related autoantibody prevalence either at diagnosis or thereafter in Sri Lankan children (9). Sensitive assays for T1D related autoantibodies identify variable percentages of antibody positive subjects at diagnosis and in established T1D, associated with regional variability, as described in a recent review (10). In subjects with established T1D, 86.8% (Europe) and 79.1% (Asia) were positive when three autoantibodies, and 92.8% (Europe) and 81.6% (Asia) were positive when four autoantibodies were assayed.

(b) predicting T1D – this is possible in high-risk individuals and in the general population. First-degree relatives of patients with T1D have a high risk of developing T1D and are obvious targets for such predictive autoantibody testing (11). Data about the prevalence of these autoantibodies in unaffected subjects in Sri Lanka are also sparse (9)

(c) understanding T1D aetiology – the variable prevalence of T1D autoantibodies in ethnically disparate groups may point to a variable role for autoimmunity in different population groups although other factors may be responsible. The coexistence of islet cell and thyroid autoimmunity in T1D has been shown previously in Sri Lankan subjects (9). However, no recent data are available.

### Aims of study

To assess in Sri Lankan T1D children and their unaffected siblings the prevalence of:

(1) GADA, IA-2A and ZnT8A, using the combined three autoantibody screening assay [Elisa RSR™ 3 Screen ICA™ (3-Screen)], confirmed by individual specific autoantibody assays.

(2) IAA, using a separate specific assay.

(3) thyroid autoantibodies to – thyroid peroxidase (TPOA), thyroglobulin (TgA) and the TSH receptor (TSHRA).

## Methods

(1) Selection of T1D subjects and their siblings –

Inclusion criteria – all children (a) with new and previously diagnosed T1D; (b) of both sexes; (c) and any age; and (d) their unaffected siblings.

Exclusion criteria – children (a) with diabetes on immunosuppressive treatment; (b) with secondary diabetes (drug induced, following pancreatitis etc); and (c) with Maturity Onset Diabetes of the Young (MODY) if known or suspected

(2) Study setting and subject recruitment – Subjects were identified from the T1D Registry of the Lady Ridgeway Hospital, Colombo 8, Sri Lanka. Informed consent was obtained from their parents or guardians. Consecutive T1D subjects and their siblings were tested during routine visits to the Diabetes Clinic. Approval was granted for this study by the Sri Lanka College of Paediatricians Ethics Committee (SLCP EC17/7/16). This study was significantly affected by the COVID pandemic and the civil unrest in the country and recruitment had to be curtailed as a result (see below)

(3) Blood sample collection and laboratory analysis - A venous blood sample was collected from subjects, centrifuged immediately and the serum frozen at -80°C. Samples were transported in dry ice to the FIRS Laboratories, RSR Ltd., Cardiff, for analysis. Samples were analyzed using 3-Screen (RSR Ltd., Cardiff, UK, https://www.rsrltd.com) and assays for GADA, IA-2A, ZnT8A and IAA (RSR Ltd., Cardiff, UK).

(4) Assay Methodology –

(a) Diabetes autoantibody ELISAs – All ELISAs used the bridging assay principle where the divalent autoantibody forms a bridge between the antigen coated onto the plates and a biotin labelled antigen added in a solution. In the assay, antigen coated plates were incubated with serum samples, calibrators and controls followed by a wash step and incubation with the antigen-biotin solution. After washing, the amount of antigen-biotin bound was determined by addition of streptavidin peroxidase and after a further wash the reaction was developed by addition of tetramethylbenzidine followed by a stop solution. The optical density (OD) of the plate wells was measured at 450 nm and 405 nm using an ELISA plate reader. Reading at 405nm allowed quantitation of high absorbances. All the ELISAs were performed following the manufacturer’s Instructions For Use (IFU, https://www.rsrltd.com).

GADA - Samples were assayed in the ELISA RSR™ GADA assay (GADA ELISA) (12). The measuring interval was 5-2000 U/mL (Units are NIBSC 97/550). The cut off ≥5.0 U/mL as indicated in the IFU was used.

IA-2A - Samples were assayed in the Elisa RSR™ IA-2A Version 2 assay (IA-2A ELISA) (13). The measuring range was 7.5-4000 U/mL (NIBSC 97/550). The cut off ≥7.5 U/mL as indicated in the IFU was used.

ZnT8A - Samples were assessed in the ELISA RSR™ ZnT8A™ assay (ZnT8A ELISA). This assay measures and detects ZnT8A specific to the 325R, 325W and residue 325 non-specific alleles (14, 15). The measuring interval was 15–2000 units/mL (arbitrary RSR units). The cut off ≥15.0 units/mL as indicated in the IFU was used.

3 Screen – In the 3 Screen ELISA the plates coated with all three antigens GADA, IA-2A and ZnT8A (15) and a mixture of all three biotinylated antigens were used. Results were expressed as an index relative to a reference preparation included in the assay and a cut off ≥30 index as indicated in the IFU was used.

RSR ELISAs’ reported sensitivity and specificity in International Autoantibody Standardization Program (IASP) workshop for 2020 were: 3 Screen (96%, 100%), GADA (88%, 98.9%), IA-2A (72%, 100%) and ZnT8A (74%, 98.9%), respectively.

(b) IAA - Samples were assessed in the RIA RSR™ IAA (IAA RIA) (16) performed following the manufacturer’s IFU (https://www.rsrltd.com). The measuring range was 0.4–50 units/mL (arbitrary RSR units). The cut off was ≥0.4 units/mL as indicated in the IFU.

(c) Thyroid Autoantibodies - TPOA and TgA (cut off > 0.3 unit/mL) were measured using RIA assays (RSR Ltd.) (17, 18). TSHRA (≥ 0.4 IU/L) were assayed in an ELISA assay (Elisa RSR™ TRAb, 3rd Generation) (19). The IFU cut offs above were used.

(5) Follow up of siblings positive for two or more T1D related antibodies -

Unaffected siblings who were positive for two or more T1D related autoantibodies were monitored with an oral glucose tolerance test (OGTT) done at the time of autoantibody detection, and they were reviewed as follows – (a) in case of normal glucose tolerance – OGTT repeated every 6 months; (b) impaired glucose tolerance – OGTT repeated every 3 months with likely early insulin treatment; (c) diabetes mellitus - referred for routine diabetes care. Parents/guardians were informed, and educational and medical support offered.

Statistical methods

Summary values are displayed as mean ± standard deviation (SD) or median (interquartile range, IQR) as appropriate. Comparisons of percentages of autoantibody prevalence between different subgroups of patients for categorical variables were made using the chi squared test. The comparison of means/medians between two groups was done using the unpaired Student’s t test and the Mann-Whitney test as appropriate (SPSS v27, IBM Corporation, USA). A statistically significant difference was defined as p< 0.05.

## Results

**Figure 1:**
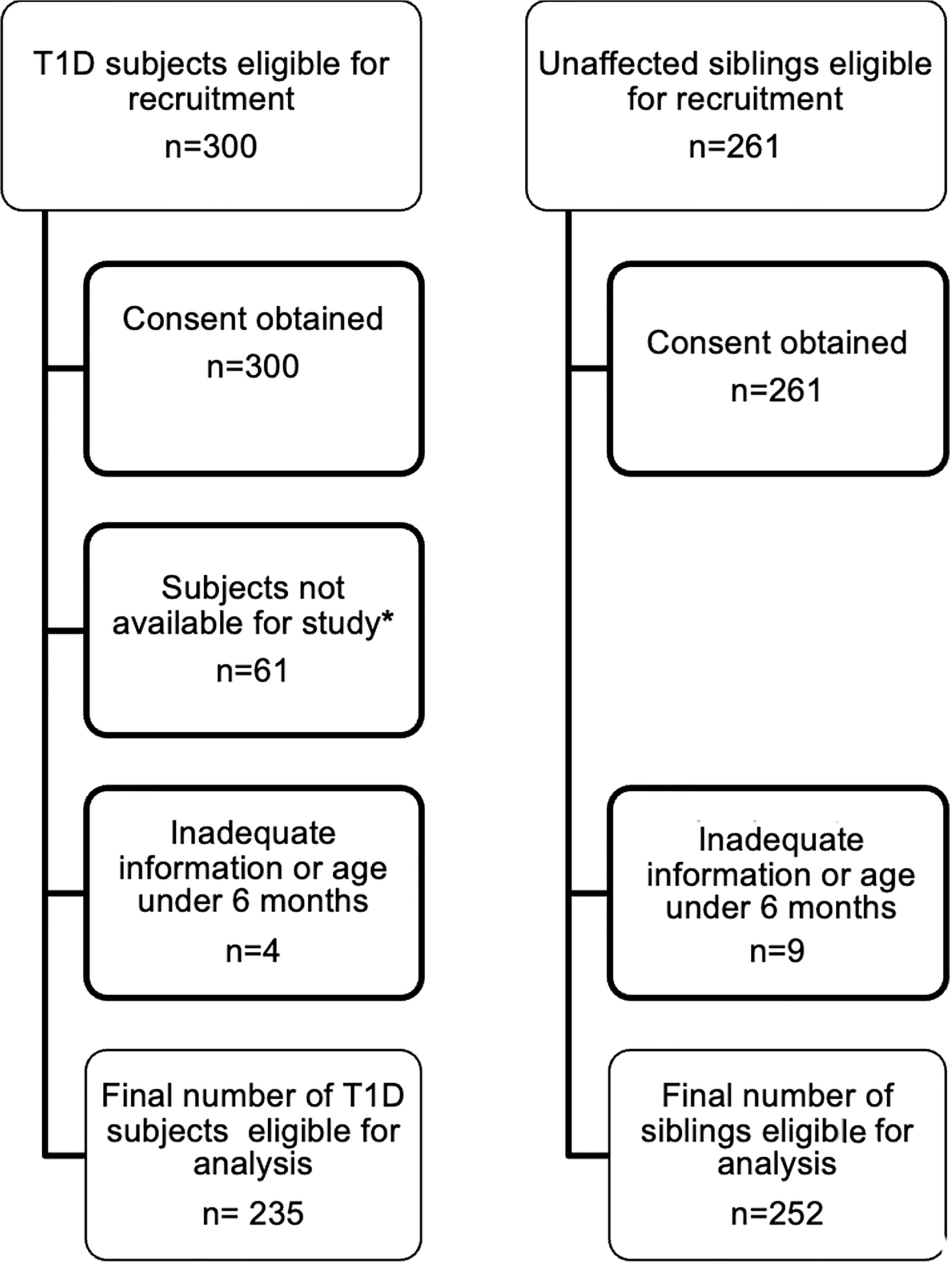
Recruitment of T1D subjects and their siblings. This flowchart describes the process of recruitment and final numbers studied *due to issues related to and disruption caused by the COVID19 pandemic and civil unrest.

Consent was obtained from parents/guardians of 300 T1D subjects and 261 siblings. However, 61 T1D subjects defaulted because of the current COVID19 pandemic, and political and civil unrest related disruption (e.g. inability to attend hospital, need for isolation, restriction of movements, lack of transport, fuel shortages, inability to transport samples abroad etc.). Also, four T1D subjects and nine siblings, with inadequate information or aged below 6 months were excluded from analysis. Samples were therefore available from 235 T1D subjects and 252 siblings (**Figure 1**; **Table 1**).

**Table 1:** Details of T1D subjects and their siblings.

T1D subjects and siblings			
	T1D subjects (n=235)	Siblings (n=252)	*P*
Median age at diagnosis (IQR)	8 years 1 month (5,5:10,5) (n=226)		
Median age at sampling (IQR)	11 years 4 months (8,4:13,2)	9 years 10 months (5,9:14,9)	
Median duration of T1D (IQR) *	23 months (7,54 months) (n=228)		
Male : Female	1:1.13	1:0.96	
Prevalence (%) of T1D related autoantibodies in 3 Screen Assay	186/235 (79.1%)	16/252 (6.3%)	0.0001
Prevalence (%) of antibodies with single specific assays	GADA – 174/235 (74%); IA2A – 73/235 (31.1%); ZnT8A – 91/235 (38.7%); IAA – 189/235 (80.4%)	GADA – 15/232 (6%); IA2A – 6/252 (2.4%); ZnT8A - 4/252 (1.6%); IAA – 6/252 (2.4%)	0.0001
Prevalence (%) of single antibodies in 3 Screen assay negative subjects	3/235 (1.3%)	0	
T1D subjects only			
	3-Screen positive	3-Screen negative	
Median age at diagnosis	7 years 8 months	10 years 1 month	0.55
Median age at sampling	10 years 7 months	14 years 3 months	0.01
Median Duration of T1D*	22 months	24 months	0.83

*82/235 (34.9%) were sampled within 12 months of diagnosis.

There was a significant difference in the prevalence of T1D related autoantibodies between T1D subjects and their siblings (p=0.0001). T1D subjects who were 3-Screen positive were significantly younger than T1D subjects who were 3-Screen negative (p=0.01).

(1) Age and duration of diabetes

(a)T1D subjects - the median age (interquartile range, IQR) of T1D subjects at diagnosis and at sampling, and the median duration of T1D (IQR) at sampling are shown in **Table 1**.

Median age at sampling was higher in 3 Screen negative T1D children (p=0.01) but there was no difference in median age at diagnosis (p=0.55) and disease duration (p=0.83) between 3 Screen positive and negative subjects (**Table 1**).

All subjects classified as antibody negative T1D were on insulin at the time of recruitment, and none was obese or had acanthosis

(b)Unaffected siblings – samples from unaffected siblings of 195 subjects with T1D were analysed (n=252). Their median age at sampling is shown in **Table 1**.

(2) Autoantibody prevalence in T1D subjects and siblings –

(a)T1D subjects

**Figure 2:**
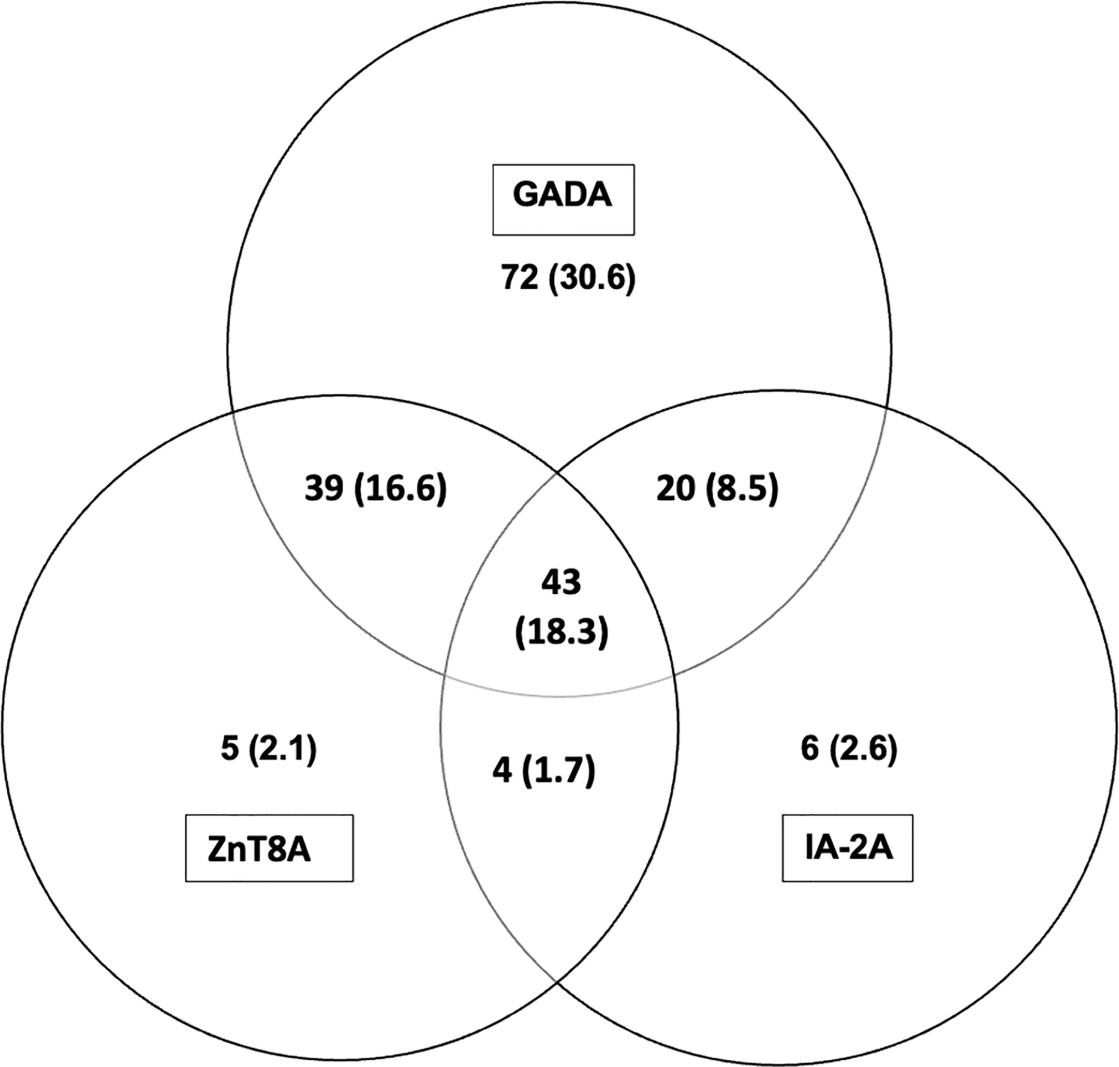
Distribution of T1D related autoantibodies in children with T1D; number and (%). A combination of all three T1D related autoantibodies were found in 18.3% and a combination of two T1D related antibodies were found in 26.8% of children with T1D (GADA + ZnT8A 16.6%; GADA + IA-2A 8.5%; ZnT8A + IA-2A 1.7%). Insulin antibodies are not included as patients were on insulin therapy before blood samples were taken.

**Figure 3:**
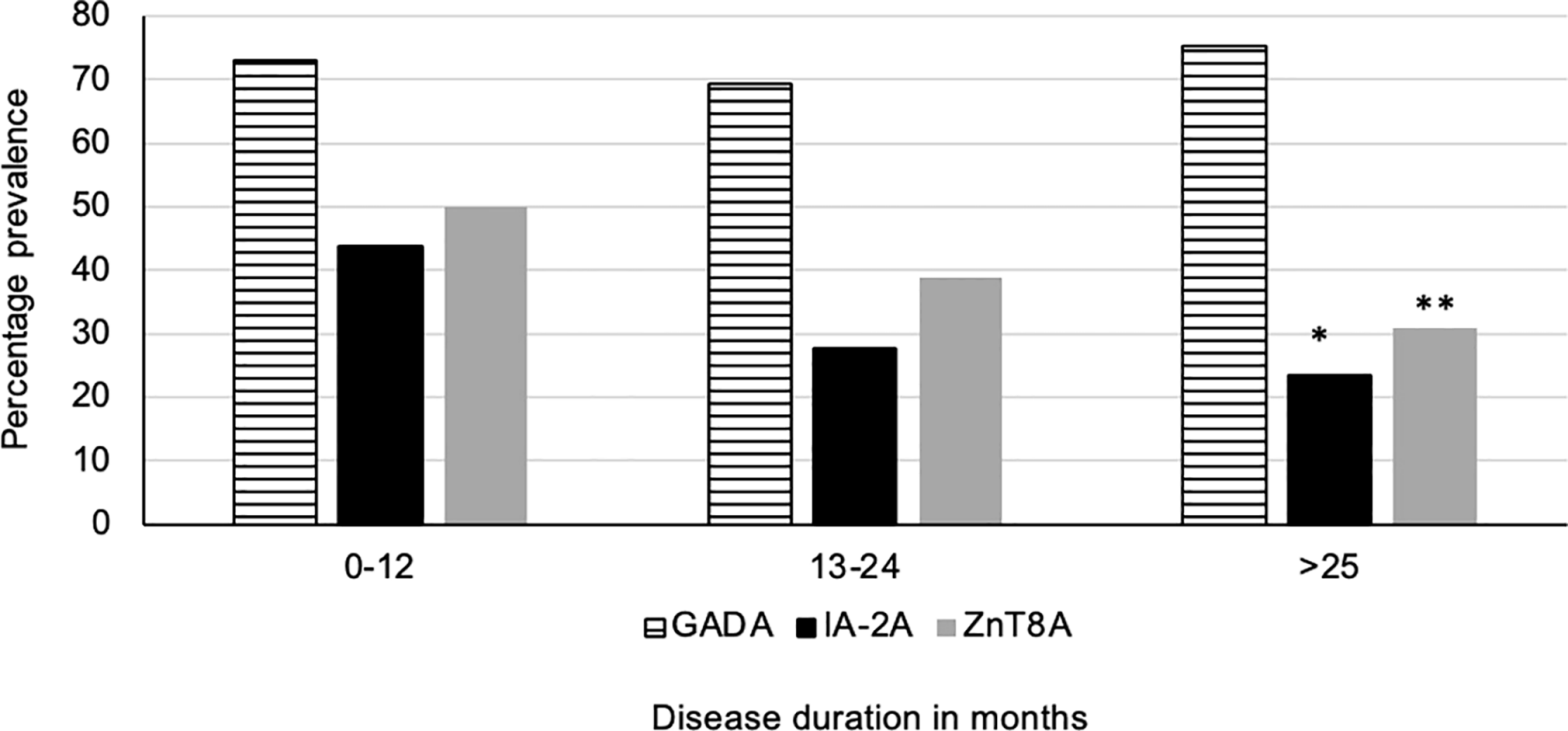
Disease duration and prevelance of T1D related autoantibodies. The prevalence of IA-2A and ZnT8A was significantly lower in children with increasing T1D disease duration (p=0.002*, p=0.006** respectively). However, despite increasing disease duration the prevalence of GADA remained stable.

(i) 186/235 (79.1%) of T1D subjects were 3-Screen positive (66.8% of them had high concentrations of antibody i.e. were ≥4 times the cut-off ≥30 index). (ii) All 186 (100%) 3-Screen positive subjects were positive for at least one component autoantibody when tested with specific assays as follows: GADA 174/235 (74%, 59.1% were ≥4 times the cut off); IA-2A 73/235 (31.1%, 22.6% ≥4 times the cut off); ZnT8A 91/235 (38.7%, 29.8% were ≥4 times the cut off) (**Table 1**). (iii) Multiple autoantibodies were found in a high proportion of 3-Screen positive subjects – a combination of three in 18.3% and two in 26.8% of subjects (**Table 2** and **Figure 2**). (iv) Insulin antibodies were present in 189/235 (80.4%) of T1D subjects – however, these were likely to be a mixture of autoantibodies and antibodies against exogenous insulin. (v) The prevalence of autoantibodies analyzed by sex, age at diagnosis, age at sampling and duration of disease are detailed in **Table 2**. (vi) The prevalence of IA-2A and ZnT8A were lower with longer disease duration (p=0.002 and p=0.006, respectively) (**Figure 3**). There was no significant difference in the distribution of GADA (p=0.89), IA-2A (p=0.96) and ZnT8A (p=0.87) between T1D males and females (**Table 2**).

Of the 49 T1D subjects who were 3 Screen negative, three had one autoantibody (one each of GADA, IA-2A or ZnT8A) detected by individual ELISAs at low concentration (≥1.25 - 2.4 times cut off). Therefore there were 46 autoantibody negative T1D subjects in total. In addition, four T1D subjects who were autoantibody negative had siblings who were T1D autoantibody positive – GADA in 2, IA-2A in 1 and IAA in 1. These T1D subjects had disease durations of 42, 6, 140 and 141 months, respectively.

(b)Unaffected siblings –

(i) The prevalence of T1D autoantibodies amongst unaffected siblings was 16/252 (6.3%) in the 3-Screen assay. (ii) All 16 were positive for at least one single autoantibody in the specific ELISAs. For the individual autoantibody ELISAs the prevalences were – 15 GADA (6.0%), six IA-2A (2.4%) and four ZnT8A (1.6%). (iii) The prevalence of IAA was 2.4% (6/252) and these were likely true insulin autoantibodies. (iv) Multiple autoantibodies were present in 4/255 (1.6%) siblings (**Supplementary Figure 1**). Importantly, four siblings had two different T1D antibodies and were followed up with oral glucose tolerance tests (OGTT) (see below) (**Supplementary Figure 1**).

A single T1D related autoantibody at low concentration was found in four 3-Screen negative unaffected siblings using single specific autoantibody tests.

**Table 2:** Autoantibody prevalence in relation to sex, age at sampling and age at onset in T1D Subjects.

	GADA (%)	IA-2A (%)	ZnT8A (%)
All T1D Samples	174/235 (74.0%)	73/235 (31.1%)	91/235 (38.7%)
Males	81/110 (73.6%)	34/110 (30.9%)	42/110 (38.2%)
Females	93/125 (74.4%)	39/125 (31.2%)	49/125 (39.2%)
Age at sampling			
7-60 months	12/18 (66.7%)	7/18 (38.9%)	9/18 (50.0%)
61 to 120 months	60/75 (80.0%)	29/75 (38.7%)	39/75 (52.0%)
121 to 180 months	90/121 (74.4%)	33/121 (27.3%)	38/121 (31.4%)
181 or more months	9/17 (53%)	3/17 (17.6%)	4/17 (23.5%)
Age at onset			
up to 60 months	37/54 (68.5%)	13/54 (24.1%)	21/54 (38.9%)
61 to 120 months	84/104 (80.8%)	44/104 (42.3%)	46/104 (44.2%)
121 or more	45/68 (66.2%)	13/68 (19.1%)	22/68 (32.3%)

(3) Follow up of autoantibody positive unaffected siblings –

The four siblings who had two T1D autoantibodies were followed up as planned. One developed impaired glucose tolerance (2-hour post OGTT plasma glucose 9.3mmo/l) and was given insulin.

Furthermore, one unaffected sibling who was only GADA positive, developed T1D four years after recruitment during a COVID19 related admission to hospital.

(4) *Thyroid autoantibody prevalence*

Samples were available for TPOA and TgA testing in 427 and TSHRA testing in 340 subjects (**Table 3**). The prevalence of TPOA (p=0.0001) and TgA (p=0.0004) were significantly higher in those with T1D compared to their siblings. In nine T1D subjects with concomitant clinical hypothyroidism TPOA and TgA were present in the majority (100%, 78% respectively). TSHRA were detected in one subject in the T1D cohort (3.3 IU/L who also had bronchial asthma and hypothyroidism) and in one subject in the sibling cohort (1.2 IU/L, who had bronchial asthma).

**Table 3:** Thyroid autoantibody prevalence T1D subjects and their siblings.

Thyroid antibody*	All T1D subjects (n=) %	T1D with Hypo (n=) %	T1D without Hypo (n=) %	Siblings (n=) %	*p*
TPOA	50 (180) – 28%	9 (9) -100%	41 (171) – 24%	11 (247) – 4.4%	0.0001**
TgA	55 (180) – 31%	7 (9) – 78%	48 (171) – 28%	39 (247) – 16%	0.0004***
TSHRA	1 (139) – 0.7%	1 (6) – 17%	0 (133)	1 (201) – 0.5%	

*Samples were insufficient to test in a proportion of T1D subjects (TPOA and TGA – 55; TSHRA 96) and siblings (TPOA and TgA – 5; TSHRA – 51); ** TPOA T1D vs siblings; *** TgA T1D vs siblings; Hypo, hypothyroidism.

## Discussion

We have shown in this cross-sectional study that the prevalence of T1D related autoantibodies in Sri Lankan children using the relatively new screening tool, 3 Screen assay, is consistent with prevalence figures for established disease in Asian countries quoted in a recent review (10). All 3-Screen positive T1D children were positive for one or more T1D antibodies when measured in individual autoantibody specific assays signifying a high correlation between 3 Screen and individual assay results (**Table 1**; **Figure 2**). The majority of 3-Screen positive children had GADA at high concentration (≥4 times the cut off) and the prevalence of IA-2A and ZnT8A declined significantly with increasing disease duration (**Figure 3**). The high IAA prevalence in them (80.4%) was likely due to antibodies against exogenous insulin. Only three (of 49) 3 Screen negative T1D children had a single autoantibody at low concentration. The 3 Screen assay is therefore an effective tool for detecting T1D related autoantibodies in T1D children, with a high correlation with specific autoantibody assays (**Table 1**). The higher prevalence of T1D related autoantibodies in those aged 61-120 months at onset and at the time of sampling, remains unexplained (**Table 2**). This is the first study using 3 Screen, to examine the prevalence of T1D related autoantibodies in Sri Lanka and should be an effective baseline for future studies. In a recent study of Bavarian children, 98% of 132 newly diagnosed T1D children were 3 Screen positive as were 91% of children in a study from Sweden (20, 21). However, the prevalence of T1D autoantibodies in this study can be compared to the mean positivity of 66% when two and 79% when three autoantibodies were tested in established T1D cases (more than 12 months duration) in Asia in the previously quoted review of 125 studies (10).

In this study, 46 T1D subjects were autoantibody negative. The reasons for autoantibody negativity in T1D may include – unidentified MODY, long disease duration, mis-classified type 2 diabetes, coexisting diseases, autoantibody reversion, ethnic differences (in particular in Asian populations), defects in antibody production, immunodysregulation etc (22, 23). We excluded subjects with a clinical suspicion of MODY. However, screening for HNF 1 and 4 alpha, HNF 4 beta or glucokinase gene mutations were not possible due to global and local situations (24). There were no reported concomitant diseases and none of the children developed diabetes under the age of 6 months making neonatal diabetes unlikely.

Unaffected siblings had a significantly lower T1D autoantibody prevalence using 3 Screen. Furthermore, 4/252 (1.6%) unaffected siblings had two T1D related autoantibodies conferring a high risk of developing T1D of 70% in 10 years in one study (25). One of these subjects developed impaired glucose tolerance and is on insulin. Furthermore, a different sibling with one T1D autoantibody also developed T1D in the course of Covid-19 infection – no samples were available for analysis in the interim period or at the time of diagnosis for this subject. The others are under surveillance. Therefore, 3-Screen is also an effective tool for detecting T1D related autoantibodies in high-risk Sri Lankan children for assessing future risk of T1D. This is the first study examining the prevalence of T1D related autoantibodies in high-risk children using 3-Screen in Sri Lanka and establishes a baseline for future studies. The above findings are different to a previous study of 122 T1D Sri Lankan subjects [median age at diagnosis of 11.2 (7.6, 14.2) and at recruitment of 16 (12,20) years] using individual ELISA assays, which demonstrated a GADA prevalence of 60.6% in T1D and 4% in unaffected subjects [median age 29 (24-35) years] (p=0.001) and IA2A of 14% vs. nil (p=0.001) respectively (9). It should be noted that this study population was older and different assay methodology was used. However, a 6.3% 3 Screen positivity in unaffected siblings in our study can be compared to an overall 7.9% multiple autoantibody and 3.5% single autoantibody positivity in 13,377 combined subjects with genetic risk of developing diabetes from DAISY, BABYDIAB, BABYDIET and DIPP studies (26). In the TEDDY study 9.5% of 7777 children developed one or more and 5.5% developed two or more diabetes autoantibodies (25).In addition, 8.0% of 1065 siblings were positive in 3 Screen in a study in Poland of whom 5.6% had multiple autoantibodies (27).

The minimum volume required to assay all autoantibodies in the study (including thyroid autoantibodies) was 410µL. However for future screening strategies a capillary blood sample of approximately 200µL should be sufficient for 3 Screen and IAA assays (20).

T1D related autoantibodies rarely occur before 6 months (28) but have a peak incidence of first appearance before 3 years (28–30). The presence of multiple T1D related autoantibodies is a significant risk for developing T1D (25). The risk is known to be higher in the 5 years after first detection, particularly in those with higher serum concentrations of IAA and IA-2A (31). T1D related autoantibodies may decline in concentration over time, and patients may become seronegative causing a decline in autoantibody prevalence (e.g. IA-2A and ZnT8A in this study, **Figure 3**) (32, 33). However the prevalence of either 1, 2 or 3 T1D autoantibodies and 3 Screen positivity does not decline with greater disease duration in our study group (data not shown here). Longitudinal measurement of T1D related autoantibodies is a good predictor of T1D in first degree relatives and in unselected populations (34). It should be noted that measurements of IAA have a greater value in screening strategies particularly in very young children and in subjects who are not treated with exogenous insulin (21).

There have been several recent studies like ours, although comparisons should be made with caution because of variable study methodology, assay differences and population characteristics (**Table 4**). In a recent global scoping review including 125 studies from 48 different countries, GADA was the most prevalent antibody in established T1D (longer than 12 months duration) followed by ZnT8A, IA-2A and IAA (10). However, there were differences in prevalence for individual antibodies across different geographical regions as mentioned earlier (10).

**Table 4:** Recent studies of T1D autoantibody prevalence in children.

Location (reference)	Size of group	Median age at onset (years)	Median duration	Median age at sampling (years)	GADA %	IA2A %	ZnT8A %
Poland (27)	114	8.2	5.2 y**	–	61	48	64
India (28)	92	–	6.3 y	14.85	79.3	32.6	20.65
Iran (29)	142	4.2	34 m***	–	56.3	40.1	–
Sri Lanka*	235	8.1	23 m	11.4	74	31	38.7

*Current study; ** y, years; *** m, months.

The prevalence of thyroid autoantibodies TPOA and TGA, were significantly higher in T1D children compared to unaffected siblings. Within this group, children who were being treated for hypothyroidism had a higher prevalence of thyroid antibodies compared to those who were not (**Table 3**). A recent study in 2507 unselected Sri Lankan children showed a prevalence of TPOA (10.3%) and TgA (6.4%) using specific age and sex derived local reference ranges as against manufacturer’s reference ranges (35). The present study had a significantly smaller number of subjects and used manufacturer’s assay cut offs, which may account for the difference. However, the prevalence of hypothyroidism amongst T1D children (5%) is broadly in agreement with other published studies (3-8%) (36). These observations attest to the well-recognised clustering of autoimmune diseases in a single individual. In particular, the combination of T1D and autoimmune thyroid disease known as the Autoimmune Polyglandular Syndrome type 3A (37).

Our study has several shortcomings – (a) the cross-sectional design with its inherent disadvantages, (b) the modest number of subjects in the two groups due to the COVID19 pandemic and local civil unrest related factors forcing the study to be discontinued prematurely, (c) the unavailability of previous studies with similar screening assay tools for comparison, (d) the lack of local age and sex derived reference ranges and (e) the absence of HLA DR and DQ allele analysis in this population – including these would add value to any future studies.

However, it also has several advantages – (a) the use of a sensitive multiple T1D autoantibody screening assay which has a good correlation with individual specific autoantibody assays, used for the first time in this population, (b) the availability of patient data in a hospital based T1D Registry, and (c) establishing prevalence data for T1D related autoantibodies using a single screening assay (3-Screen) both in T1D children and their unaffected siblings for the first time in Sri Lanka.

## Conclusions

We have demonstrated the prevalence of T1D related autoantibodies in Sri Lanka in both T1D subjects and their unaffected siblings using a screening tool, 3-Screen. This assay is a valuable tool for screening T1D subjects and high-risk groups, showing a high correlation with individual specific autoantibody assays. Our study forms the basis for future investigations of autoantibody prevalence and T1D prediction models in the Sri Lankan population.

## Data availability statement

The original contributions presented in the study are included in **Supplementary Materials**. Further inquiries can be directed to the corresponding author.

## Ethics statement

The studies involving human participants were reviewed and approved by Sri Lanka College of Paediatricians Colombo. Written informed consent to participate in this study was provided by the participants’ legal guardian/next of kin.

## Author contributions

NA, DdS, KdS, LDP conceived and developed the idea for the study and were involved in the planning of it. MP, JF, BRS also contributed to the study design. NA did the clinical investigations, and MP, JF, BRS contributed to analysing the data and writing the paper. MA performed the autoantibody assays and data analysis. All authors contributed to the article and approved the submitted version.

## References

[1] Atkinson MA Eisenbarth GS Michels AW. Type 1 diabetes. Lancet (2014) 383:69–82. doi: 10.1016/S0140-6736(13)60591-7 23890997 PMC4380133

[2] Eisenbarth GS. Type I diabetes mellitus. a chronic autoimmune disease. N Engl J Med (1986) 314:1360–8. doi: 10.1056/NEJM198605223142106 3517648

[3] Redondo MJ Eisenbarth GS. Genetic control of autoimmunity in type I diabetes and associated disorders. Diabetologia (2002) 45:605–22. doi: 10.1007/s00125-002-0781-1 12107741

[4] Patterson CC Dahlquist GG Gyürüs E Green A Soltész G. Incidence trends for childhood type 1 diabetes in Europe during 1989-2003 and predicted new cases 2005-20: a multicentre prospective registration study. Lancet (2009) 373:2027–33. doi: 10.1016/S0140-6736(09)60568-7 19481249

[5] Karvonen M Viik-Kajander M Moltchanova E Libman I LaPorte R Tuomilehto J. Incidence of childhood type 1 diabetes worldwide. diabetes mondiale (DiaMond) project group. Diabetes Care (2000) 23:1516–26. doi: 10.2337/diacare.23.10.1516 11023146

[6] Atapattu N de Silva KSH. Improving diabetes care in Sri Lankan children: the way forward. Sri Lanka J Diabetes Endocrinol Metab (2012) 2:35–8. doi: 10.4038/sjdem.v2i1.4333

[7] Lampasona V Liberati D. Islet autoantibodies. Curr Diab Rep (2016) 16:53. doi: 10.1007/s11892-016-0738-2 27112957

[8] Ilonen J Lempainen J Veijola R. The heterogeneous pathogenesis of type 1 diabetes mellitus. Nat Rev Endocrinol (2019) 15:635 – 50. doi: 10.1038/s41574-019-0254-y 31534209

[9] Premawardhana LDKE Wijeyaratne CN Chen S Wijesuriya M Illangasekara U Brooking H. Islet cell, thyroid, adrenal and celiac disease related autoantibodies inpatients with type 1 diabetes from Sri Lanka. J Endocrinol Invest (2006) 29:968–74. doi: 10.1007/BF03349209 17259793

[10] Ross C Warad ZJ Gomber A Owais M Yeh JM Reddy C-L Atun R. The prevalence of islet autoantibodies in children and adolescents with type 1 diabetes mellitus: a global scoping review. Front Endocrinol (2022) 13:2022.815703. doi: 10.3389/fendo.2022.815703 PMC8851309 35185797

[11] Ziegler AG Nepom GT. Age related islet antibody incidence in the offspring of patients with type 1 diabetes. Diabetologia (2012) 55:1937–43. doi: 10.1007/s00125-012-2472-x 22289814

[12] Brooking H Ananieva-Jordanova R Arnold C Amoroso M Powell M Betterle C. A sensitive non-isotopic assay for GAD65 autoantibodies. Clin Chim Acta (2003) 331:55–9. doi: 10.1016/S0009-8981(03)00088-3 12691864

[13] Chen S Willis J Maclean C Ananieva-Jordanova R Amoroso MA Brooking H. Sensitive non-isotopic assays for autoantibodies to IA-2 and to a combination of both IA-2 and GAD65. Clin Chim Acta (2005) 357:74–83. doi: 10.1016/j.cccn.2005.02.006 15963796

[14] Petruzelkova L Ananieva-Jordanova R Vcelakova J Vesely Z Stechova K Lebl J. The dynamic changes of zinc transporter 8 autoantibodies in Czech children from the onset of type 1 diabetes mellitus. Diabetes Med (2014) 2014:31165–71. doi: 10.1111/dme.12308 23952619

[15] Dunseath G Ananieva-Jordanova R Coles R Powell M Amoroso M Furmaniak J. Bridging-type enzyme-linked immunoassay for zinc transporter 8 autoantibody measurements in adult patients with diabetes mellitus. Clin Chim Acta (2015) 447:90–5. doi: 10.1016/j.cca.2015.05.010 26006309

[16] Masuda M Powell M Chen S Beer C Fichna P Rees Smith B. Autoantibodies to IA-2 in insulin-dependent diabetes mellitus. measurements with a new immunoprecipitation assay. Clin Chim Acta (2000) 291:53–66. doi: 10.1016/S0009-8981(99)00199-0 10612717

[17] Prentice LM Phillips DI Sarsero D Beever K McLachlan SM Smith BR. Geographical distribution of subclinical autoimmune thyroid disease in Britain: a study using highly sensitive direct assays for autoantibodies to thyroglobulin and thyroid peroxidase. Acta Endocrinol (Copenh) (1990) 123:493–8. doi: 10.1530/acta.0.1230493 2256432

[18] Beever K Bradbury J Phillips D McLachlan SM Pegg C Goral A. Highly sensitive assays of autoantibodies to thyroglobulin and to thyroid peroxidase. Clin Chem (1989) 35:1949–54. doi: 10.1093/clinchem/35.9.1949 2776323

[19] Smith BR Bolton J Young S Collyer A Weeden A Bradbury J. A new assay for thyrotropin receptor autoantibodies. Thyroid (2004) 14:830–5. doi: 10.1089/1050725042451248 15588379

[20] Amoroso M Achenbach P Powell M Coles R Chlebowska M Carr L. 3 screen islet cell autoantibody ELISA: A sensitive and specific ELISA for the combined measurement of autoantibodies to GAD_65_, to IA-2 and to ZnT8. Clin Chim Acta (2016) 462:60–4. doi: 10.1016/j.cca.2016.08.013 27570064

[21] Törn C Vaziri-Sani F Ramelius A Larsson HE Ivarsson SA Amoroso M. Evaluation of the RSR 3 screen ICA™ and 2 screen ICA™ as screening assays for type 1 diabetes in Sweden Acta Diabetologica (2022) 59:773–81. doi: 10.1007/s00592-022-01856-5 PMC9085662 35220476

[22] Pozzilli P Visalli N Leslie D. No evidence of rapid onset (Japanese) of type 1 diabetes in Caucasian patients. IMDIAB group. Diabetologia (2000) 43:1322. doi: 10.1007/pl00022647 11079755

[23] Balasubramanian K Dabadghao P Bhatia V Colman PG Gellert SA Bharadwaj U. High frequency of type 1B (idiopathic) diabetes in north Indian children with recent-onset diabetes -. Diabetes Care (2003) 26:2697. doi: 10.2337/diacare.26.9.2697 12941746

[24] Hattersley AT. Maturity-onset diabetes of the young: clinical heterogeneity explained by genetic heterogeneity. Diabetic Med (1998) 15:15–24. doi: 10.1002/(SICI)1096-9136(199801)15:1<15::AID-DIA562>3.0.CO;2-M 9472859

[25] Krischer JP Lynch KF Schatz DA Ilonen J Lernmark Å Hagopian WA. The 6-year incidence of diabetes-associated autoantibodies in genetically at-risk children: the TEDDY study. Diabetologia (2015) 58:980–7. doi: 10.1007/s00125-015-3514-y PMC4393776 25660258

[26] Ziegler AG Rewers M Simell O Simell T Lempainen J Steck A. Seroconversion to multiple islet autoantibodies and risk of progression to diabetes in children. JAMA (2013) 309:2473–9. doi: 10.1001/jama.2013.6285 PMC4878912 23780460

[27] Bossowski A Noiszewska K Polkowska A Zasim A Mysliwiec M Szadkowska A. £ screen ICA ELISA – a new tool to identify pre-clinical diabetes in first-degree relatives of patients with type 1 diabetes (pre-d1abetes study). Pediatr Diabetes (2022) 23:150.34773333

[28] Parikka V Näntö-Salonen K Saarinen M Simell T Ilonen J Hyöty H. Early seroconversion and rapidly increasing autoantibody concentrations predict prepubertal manifestation of type 1 diabetes in children at genetic risk. Diabetologia (2012) 55:1926–36. doi: 10.1007/s00125-012-2523-3 22441569

[29] Primavera M Giannini M Chiarelli F. Prediction and prevention of type 1 diabetes. Front Endocrinol (2020) https:248. doi: 10.3389/fendo.2020.00248 PMC7326081 32670194

[30] Knip M Korhonen S Kulmala P Veijola R Reunanen A Raitakari OT. Prediction of type 1 diabetes in the general population. Diabetes Care (2010) 33:1206–12. doi: 10.2337/dc09-1040 PMC2875424 20508230

[31] Bonifacio E Achenbach P. Birth and coming of age of islet autoantibodies. Clin Exper Immunol (2019) 198:294–305. doi: 10.1111/cei.13360 31397889 PMC6857083

[32] So M Speake C Steck AK Lundgren M Colman PG Palmer JP. Advances in type 1 diabetes prediction using islet autoantibodies: Beyond a simple count. Endocrine Rev (2021) 42:584–604. doi: 10.1210/endrev/bnab013 33881515

[33] Głowińska-Olszewska B Michalak J Łuczyński W Larosa M Chen S Furmaniak J. Organ-specific autoimmunity in relation to clinical characteristics in children with long-lasting type 1diabetes. J Pediatr Endocrinol Metab (2016) 29:647–56. doi: 10.1515/jpem-2015-0190 27008690

[34] Basu M Pandit K Banerjee M Mondal S Mukhopadhyay P Ghosh S. Profile of autoantibodies (Disease related and other) in children with type 1 diabetes. Indian J Endocrinol Metab (2020) 24:256–9. doi: 10.4103/ijem.IJEM_63_20 PMC7539037 33083265

[35] Jayatissa R Okosieme O.E. Ranasinghe S JL C Gunatunge I Lazarus JH Premawardhana LD. Thyroid autoimmunity and dysfunction in Sri Lankan children and adolescents after 22 years of sustained universal salt iodization. Thyroid (2021) 31:1103–13. doi: 10.1089/thy.2020.0798 33406977

[36] Mahmud FH Elbarabry NS Reiterer E Holl RW Kordonouri O Knip M. ISPAD clinical practice guidelines 2018: Other associated complications and conditions in children and adolescents with type 1 diabetes. Paediatr Diabetes (2018) 19:275–86. doi: 10.1111/pedi.12740 PMC6748835 30066458

[37] Betterle C Sabbadin C Scaroni C Presotto F. Colao AM Jaffrain-Rea ML Beckers A, editors. Polyendocrine disorders and endocrine neoplastic syndromes. Switzerland AG: Endocrinology Springer Nature (2019). p. 1–50. doi: 10.1007/978-3-319-73082

