# Supplementary material for: The prevalence of diabetes and thyroid related autoantibodies in Sri Lankan children with type 1 diabetes and their unaffected siblings – The utility of a new screening assay
Source: Front Endocrinol (Lausanne). 2023 Feb 6;14:1028285. doi: 10.3389/fendo.2023.1028285 (PMC9939822; doi:10.3389/fendo.2023.1028285)
Supplement: Supplementary file 1 [file DataSheet_1.pdf]

	Ref No	T1D or sibling	Age at sampling month	Duration of T1D month	Sex	3 Screen ICA ELISA Index
						Cut off Index ≥30.0
1	1	T1D	123	3	Male	980.0
2	2	Sibling	84		Male	8.2
3	3	T1D	136	42	Male	8.7
4	4	Sibling	102		Female	19.7
5	5	T1D		37	Male	776.0
6	6	Sibling	110		Female	6.9
7	7	T1D	148	41	Male	935.5
8	8	Sibling	104		Male	7.2
9	9	Sibling	30		Male	7.2
10	10	T1D	179	64	Female	27.0
11	11	Sibling	150		Female	7.0
12	12	Sibling	106		Male	7.0
13	13	Sibling	55		Male	6.9
14	14	T1D	173	48	Female	124.5
15	15	Sibling	143		Female	7.9
16	16	T1D	39	12	Female	919.5
17	17	Sibling	198		Female	7.9
18	18	Sibling	114		Male	6.9
19	19	T1D	127	79	Female	159.2
20	20	Sibling	50		Male	7.9
21	21	T1D	140	9	Female	1487.4
22	22	Sibling	35		Male	7.7
23	23	Sibling	181		Female	7.0
24	24	T1D	155	21	Female	351.2
25	25	Sibling	117		Female	7.4
26	26	Sibling	20		Male	7.4
27	27	T1D	129	15	Female	385.8
28	28	Sibling	154		Female	9.1
29	29	Sibling	30		Female	9.4
30	30	T1D	101	33	Male	81.7
31	31	Sibling	50		Female	8.4
32	32	Sibling	147		Male	7.9
33	33	T1D	90	23	Female	479.1
34	34	Sibling	105		Female	1048.1
35	35	Sibling	38		Male	8.1
36	36	T1D	97	15	Female	1291.4
37	37	Sibling	73		Male	8.9
38	38	T1D	96	47	Female	199.8
39	39	Sibling	140		Male	252.0
40	40	Sibling	188		Male	6.0
41	41	T1D	128	44	Female	726.8
42	42	Sibling	41		Male	6.7
43	43	T1D	104	6	Female	24.7
44	44	Sibling	82		Male	819.2
45	45	Sibling	45		Male	6.3
46	46	T1D	57	28	Male	54.6
47	47	Sibling	101		Male	5.5
48	48	T1D	119	64	Female	1011.4

49	49	Sibling	214		Female	7.3
50	50	Sibling	93		Female	8.8
51	51	T1D	165	76	Male	394.6
52	52	Sibling	129		Female	7.2
53	53	T1D	166		Female	1011.8
54	54	T1D	54	131	Male	804.9
55	55	T1D	123	2	Female	1046.5
56	56	Sibling	123		Female	844.1
57	57	T1D	159	24	Male	153.1
58	58	Sibling	114		Female	8.2
59	59	Sibling	46		Male	6.4
60	60	T1D	93	54	Male	591.7
61	61	Sibling	161		Male	6.9
62	62	T1D	152	55	Female	719.2
63	63	Sibling	72		Female	5.7
64	64	T1D	136	18	Female	65.8
65	65	Sibling	217		Female	7.0
66	66	T1D	138	16	Female	7.5
67	67	Sibling	155		Male	6.3
68	68	T1D	93	39	Male	141.1
69	69	Sibling	57		Female	7.3
70	70	T1D	133	66	Male	30.9
71	71	Sibling	69		Male	5.4
72	72	T1D	156	42	Female	401.8
73	73	Sibling	75		Female	682.6
74	74	T1D	137	49	Male	183.5
75	75	Sibling	98		Male	14.0
76	76	Sibling	44		Female	6.5
77	77	T1D	92	56	Male	15.3
78	78	Sibling	167		Male	6.5
79	79	T1D	153	11	Female	11.0
80	80	Sibling	94		Female	7.0
81	81	Sibling	24		Male	6.8
82	82	T1D	163	86	Male	354.2
83	83	Sibling	125		Male	158.1
84	84	Sibling	207		Male	7.3
85	85	T1D	151	27	Male	645.6
86	86	T1D	124	67	Female	275.4
87	87	Sibling	124		Female	6.7
88	88	T1D	75	10	Female	658.7
89	89	Sibling	49		Male	53.9
90	90	T1D	158	16	Female	11.9
91	91	T1D	93	3	Male	1007.3
92	92	Sibling	164		Male	53.8
93	93	T1D	157	39	Female	63.3
94	94	Sibling	93		Female	8.6
95	95	T1D	162	39	Male	524.9
96	97	Sibling	53		Male	7.3
97	98	T1D	161	17	Female	711.9
98	99	Sibling	229		Female	7.0
99	100	Sibling	105		Female	15.2
100	101	T1D	139	22	Male	1149.4

101	102	Sibling	254		Female	12.2
102	103	Sibling	235		Male	11.5
103	104	T1D	121	18	Female	1215.5
104	105	Sibling	173		Male	14.4
105	106	T1D	117	96	Female	12.0
106	107	Sibling	234		Female	13.2
107	108	Sibling	201		Female	8.8
108	109	T1D	114	3	Female	668.3
109	110	Sibling	38		Female	9.1
110	111	T1D	57	128	Female	53.9
111	112	Sibling	89		Male	9.6
112	113	Sibling	111		Male	9.5
113	114	T1D	158	17	Female	1315.5
114	115	Sibling	130		Female	11.9
115	116	T1D	147	50	Female	840.3
116	117	Sibling	67		Female	10.1
117	118	T1D	55	4	Male	8.9
118	119	T1D	48	20	Male	91.4
119	120	T1D	123	28	Female	46.5
120	121	Sibling	15		Male	10.7
121	122	Sibling	128		Female	9.6
122	123	T1D	102	8	Female	641.3
123	124	Sibling	170		Male	11.7
124	125	Sibling	26		Female	11.3
125	126	T1D	148		Male	131.7
126	127	Sibling	127		Male	10.1
127	128	Sibling	55		Female	27.0
128	129	T1D	131	63	Male	937.5
129	130	T1D	119	9	Female	9.6
130	131	T1D	155	39	Female	658.9
131	132	Sibling	212		Female	9.8
132	133	T1D	144	8	Male	441.0
133	134	Sibling	120		Female	15.1
134	135	T1D	200	112	Female	10.8
135	136	Sibling	246		Male	10.0
136	137	T1D	163	120	Male	25.6
137	138	T1D	68	24	Male	37.5
138	140	T1D	83	54	Female	16.2
139	141	Sibling	100		Male	9.3
140	142	T1D	158	31	Male	864.0
141	143	Sibling	209		Male	11.5
142	144	Sibling	189		Female	9.1
143	145	T1D	179	36	Female	599.4
144	146	Sibling	88		Female	916.7
145	147	T1D	136	52	Male	229.1
146	148	Sibling	150		Female	9.4
147	149	T1D	214	92	Female	277.5
148	150	Sibling	98		Female	8.5
149	151	T1D	146	74	Male	44.6
150	152	Sibling	168		Male	8.1
151	153	Sibling	214		Male	8.1
152	154	T1D	189	57	Male	8.8

153	155	Sibling	211		Male	8.1
154	156	Sibling	98		Female	8.1
155	157	T1D	155	3	Female	7.6
156	158	Sibling	210		Male	8.4
157	159	T1D	56	8	Male	93.9
158	160	Sibling	129		Female	8.4
159	161	Sibling	84		Male	9.3
160	165	T1D	146		Male	770.1
161	166	Sibling	206		Female	9.0
162	167 A	T1D	147	17	Female	1056.9
163	167 B	T1D	143	96	Female	131.6
164	168	Sibling	59		Male	8.2
165	169	T1D	158	14	Male	617.2
166	170	T1D	77	53	Female	11.2
167	171	Sibling	77		Male	8.2
168	172	T1D	105	62	Male	593.0
169	173	Sibling	116		Female	8.1
170	174	T1D	147	12	Female	1052.1
171	175	Sibling	200		Male	10.4
172	176	T1D	174	67	Male	819.5
173	177	Sibling	134		Male	9.3
174	178	Sibling	108		Female	11.3
175	179	T1D	185	134	Female	10.0
176	180	T1D	124	40	Male	10.6
177	181	T1D	86	16	Male	95.6
178	182	Sibling	142		Male	9.0
179	183	T1D	174	57	Female	118.0
180	184	Sibling	138		Female	9.3
181	185	Sibling	113		Male	11.2
182	186	Sibling	64		Female	10.9
183	187	T1D	150	96	Male	1078.6
184	188	Sibling	124		Male	973.9
185	189	T1D	95	26	Female	346.0
186	190	Sibling	66		Male	12.5
187	191	T1D	96	19	Male	881.6
188	192	Sibling	116		Female	13.6
189	193	Sibling	36		Female	16.4
190	194	T1D	164	6	Male	840.4
191	197	T1D	141	37	Male	1252.2
192	198	Sibling	110		Male	726.1
193	199	Sibling	84		Male	11.2
194	200	Sibling	54		Female	10.3
195	201	T1D	112	1	Male	1238.6
196	202	Sibling	96		Male	12.7
197	203	T1D	122	5	Male	1122.2
198	204	T1D	86	21	Male	1004.3
199	205	Sibling	22		Female	4.3
200	206	T1D	138	2 days	Male	978.5
201	207	Sibling	64		Female	3.8
202	208	T1D	86	3	Male	1153.3
203	209	Sibling	196		Male	4.8
204	210	T1D	160	154	Female	48.1

205	211	Sibling	241		Female	4.0
206	212	T1D	53	23	Female	639.7
207	213	T1D	122	43	Male	68.0
208	214	Sibling	156		Male	28.7
209	215	T1D	97	28	Male	5.3
210	216	Sibling	138		Male	18.6
211	218	T1D	190	41	Female	6.4
212	219	T1D	201		Female	31.6
213	220	T1D	82	7	Female	298.6
214	221	T1D	172	27	Male	797.4
215	222	Sibling	146		Female	5.7
216	223	T1D	125	92	Female	108.8
217	224	Sibling	152		Female	4.0
218	225	T1D	186	108	Female	341.1
219	226	Sibling	166		Male	6.4
220	227	T1D	161	96	Male	77.8
221	228	Sibling	248		Female	6.0
222	229	T1D	183	30	Female	1149.1
223	230	Sibling	208		Male	4.5
224	231	T1D	91	31	Female	636.8
225	232	Sibling	166		Male	8.8
226	233	Sibling	240		Male	5.3
227	234	T1D	62	4	Female	753.6
228	235	Sibling	39		Female	2.9
229	236	T1D	87	62	Female	220.6
230	237	T1D	121	26	Female	14.6
231	238	Sibling	86		Female	4.1
232	239	T1D	93	18	Male	830.8
233	240	Sibling	40		Female	7.0
234	241	Sibling	22		Female	3.8
235	242	T1D	116	86	Male	1247.6
236	243	Sibling	12		Female	5.4
237	244	T1D	150	1	Female	4.7
238	245	Sibling	86		Female	8.7
239	246	T1D	155	3	Male	306.4
240	247	Sibling	71		Male	8.8
241	248	T1D	64	11	Male	303.3
242	249	T1D	100	11 days	Male	1191.3
243	250	Sibling	136		Female	4.6
244	251	T1D	59	2	Male	624.4
245	252	T1D	184	24	Male	5.9
246	253	T1D	23	1	Male	943.2
247	254	Sibling	171		Male	8.7
248	255	Sibling	70		Male	5.7
249	256	T1D	177	74	Female	788.5
250	257	Sibling	139		Female	6.2
251	258	Sibling	50		Female	6.5
252	259	T1D	125	48	Male	52.9
253	260	Sibling	70		Male	5.6
254	261	T1D	152	2	Female	5.7
255	262	Sibling	281		Male	4.4
256	263	Sibling	205		Female	4.4

264	T1D	64	6	Female	1160.2
265	T1D	180	140	Male	5.4
266	Sibling	158		Male	64.8
267	T1D	141	20	Male	1053.4
268	Sibling	197		Male	4.1
269	T1D	111	43	Male	690.0
270	T1D	46	1	Female	916.5
271	T1D	113	42	Female	25.2
272	T1D	181	71	Male	159.6
273	T1D	115	60	Female	301.1
274	T1D	62	1	Female	203.6
275	T1D	187	46	Female	9.0
276	T1D	158	36	Male	413.3
277	Sibling	199		Male	5.1
278	T1D	161	32	Female	780.7
279	Sibling	209		Male	6.8
280	Sibling	41		Male	7.6
281	T1D	150	48	Female	114.9
282	Sibling	129		Male	3.9
283	Sibling	178		Female	3.6
284	T1D	154	1	Male	3.7
285	Sibling	191		Male	3.7
286	T1D	160	1	Male	1059.1
287	Sibling	28		Female	4.9
288	T1D	180	18	Male	4.4
289	Sibling	192		Female	9.5
290	T1D	167	96	Male	13.5
291	Sibling	199		Female	5.6
292	Sibling	242		Male	4.7
293	Sibling	104		Female	4.4.
294	T1D	164	1	Male	3.6.
295	T1D	153	38	Male	10.7
296	Sibling	234		Female	4.6
297	T1D	81	3	Female	848.1
298	Sibling	164		Male	3.9
299	T1D	138	90	Female	1098.3
300	Sibling	193		Female	3.7
301	T1D	178	10	Female	4.1
302	Sibling	227		Female	17.5
303	T1D	166	40	Male	66.5
304	Sibling	144		Male	4.6
305	T1D	205	62	Male	10.4
306	Sibling	142		Female	6.3
307	T1D	146	9	Female	1088.0
308	Sibling	171		Female	4.7
309	T1D	164	28	Female	65.7
310	Sibling	178		Male	5.8
311	T1D	14	13	Female	852.9
312	Sibling	93		Male	4.7
313	T1D	37	16	Male	150.7
316	T1D	192	64	Female	616.9
317	Sibling	268		Male	5.4

309	318	T1D	129	44	Female	958.0
310	319	T1D	191	37	Male	821.9
311	320	Sibling	148		Male	4.4
312	321	T1D	115	20	Male	813.4
313	322	Sibling	9		Female	7.7
314	323	Sibling	28		Male	5.4
315	324	T1D	114	42	Male	707.2
316	325	Sibling	142		Male	68.7
317	326	T1D	118	1	Male	888.3
318	327	T1D	115	1	Male	635.0
319	328	Sibling	176		Male	5.4
320	329	T1D	29	7	Male	857.2
321	330	T1D	57	12	Male	883.0
322	331	T1D	81	3	Female	1021.1
323	332	T1D	105	65	Male	41.6
324	333	T1D	175	95	Male	258.9
325	334	T1D	158	4	Female	1325.7
326	335	Sibling	303		Female	4.7
327	336	Sibling	195		Female	4.9
328	337	T1D	87	16	Female	351.5
329	338 - A	Sibling	101		Male	4.3
330	338 - B	Sibling	273		Male	5.5
331	339	Sibling	160		Male	5.2
332	340	T1D	203	96	Female	1017.7
333	341	Sibling	185		Female	14.1
334	342	T1D	164	4	Female	1087.8
335	343	Sibling	148		Female	4.1
336	344	T1D	162	15	Male	976.8
337	345	Sibling	98		Female	4.1
338	346	Sibling	91		Male	4.3
339	347	T1D	151	104	Female	18.9
340	348	T1D	107	72	Male	4.3
341	349	Sibling	151		Female	6.9
342	350	Sibling	120		Female	3.8
343	351	T1D	80	11	Male	485.9
344	352	T1D	88	3	Female	1177.1
345	353	Sibling	125		Male	5.5
346	354	T1D	31	5	Female	196.7
347	355	Sibling	61		Female	4.1
348	356	T1D	155	25	Female	773.5
349	357	Sibling	108		Male	6.2
350	358	T1D	103	3	Male	72.2
351	359	Sibling	85		Female	5.5
352	360	Sibling	162		Female	6.2
353	361	T1D	131	1	Male	4.5
354	366	T1D	111	38	Male	467.0
355	367	Sibling	61		Male	4.8
356	368	T1D	140	13	Male	344.8
357	369	Sibling	93		Male	6.9
358	370	Sibling	186		Female	13.9
359	371	T1D	145	3	Male	493.0
360	372	Sibling	65		Female	5.3

373	T1D	153	5	Female	5.0
374	Sibling	273		Female	4.5
375	Sibling	199		Male	4.5
376	T1D	211	2	Female	91.2
377	Sibling	142		Female	6.0
378	T1D	116	5	Female	837.4
379	Sibling	62		Male	6.8
380	T1D	83	35	Male	24.1
381	Sibling	16		Female	7.3
382	T1D	163	42	Female	8.3
383	Sibling	246		Female	6.0
384	T1D	121	1	Male	942.5
385	T1D	155	34	Female	1022.3
386	Sibling	293		Male	63.6
387	T1D	145	11	Female	6.1
388	Sibling	216		Female	10.6
389	Sibling	194		Male	5.5
390	T1D	115	143	Male	784.0
391	Sibling	89		Female	6.0
392	Sibling	167		Female	7.4
393	T1D	142	46	Male	1006.4
394	Sibling	202		Female	8.6
395	T1D	182	138	Female	6.0
396	Sibling	89		Female	8.8
398	T1D	51	126	Female	294.7
399	Sibling	166		Female	6.6
400	T1D	116	51	Female	905.3
401	Sibling	159		Female	6.1
402	Sibling	164		Male	977.7
403	T1D	97	58	Male	251.0
404	Sibling	26		Male	5.9
405	Sibling	176		Female	27.4
406	T1D	100	64	Female	363.3
407	T1D	61	7	Female	823.2
408	Sibling	20		Male	7.0
409	T1D	77	1	Female	707.7
410	Sibling	20		Male	5.3
411	T1D	107	70	Female	39.0
412	Sibling	141		Male	5.2
413	Sibling	25		Female	5.4
414	T1D	54	14	Female	8.3
415	T1D	146	29	Male	204.7
416	Sibling	185		Male	4.9
417	Sibling	107		Female	10.2
418	Sibling	62		Male	4.9
419	T1D	122	17	Female	989.8
420	Sibling	57		Male	4.6
421	T1D	164	12	Female	897.7
422	Sibling	65		Male	52.4
423	T1D	120	1	Male	57.2
424	Sibling	226		Male	5.0
425	T1D	149	2	Male	916.8

426	T1D	140	37	Male	79.1
427	Sibling	192		Male	6.6
428	T1D	135	12	Female	933.2
429	Sibling	59		Male	4.6
430	T1D	143	29	Female	582.5
431	T1D	152	1	Female	10.5
434	T1D	47	3	Female	103.9
435	Sibling	211		Male	12.5
436	T1D	109	19	Male	285.8
437	Sibling	88		Male	4.9
438	Sibling	71		Female	5.9
439	T1D	118	8	Female	768.6
440	Sibling	34		Female	4.9
441	Sibling	191		Female	4.6
442	T1D	137	13	Female	8.6
443	T1D	117	9	Male	442.3
444	T1D	152	72	Female	423.4
445	T1D	97	4	Female	103.4
446	Sibling	158		Male	5.6
447	Sibling	218		Female	14.1
448	Sibling	237		Female	4.1
449	T1D	158	1	Female	631.2
450	T1D	107	1	Male	667.5
451	Sibling	147		Male	4.7.
452	T1D	167	1	Male	680.5
453	Sibling	94		Female	6.7.
454	Sibling	206		Female	4.4
455	T1D	151	67	Female	1096.0
456	Sibling	53		Male	11.5
457	T1D	125	30	Female	992.3
458	T1D	169	60	Female	253.6
459	Sibling	119		Female	22.6
461	Sibling	35		Male	12.9
462	Sibling	56		Male	8.3
463	T1D	104	16	Male	313.0
464	T1D	161	50	Female	254.0
465	Sibling	119		Male	11.7
466	T1D	163	1	Male	815.8
468	T1D	118	57	Female	1154.7
469	Sibling	14		Male	10.8
470	T1D	159	72	Male	990.4
471	Sibling	113		Female	10.4
472	Sibling	80		Male	6.5
473	Sibling	43		Male	7.7
474	Sibling	21		Female	9.0
475	Sibling	236		Male	10.2
476	T1D	141	15	Male	16.0
478	T1D	139	1	Female	1016.0
479	Sibling	43		Female	4.4.
480	Sibling	37		Female	18.4
481	Sibling	81		Female	5.0
482	T1D	72	42	Female	164.4

483	Sibling	19		Male	6.0
484	T1D	120	5	Male	872.3
485	Sibling	248		Female	6.0
486	Sibling	11		Male	6.7
487	T1D	135	2	Female	8.1
488	T1D	21	2	Male	15.3
489	Sibling	93		Female	6.7
490	T1D	114	2	Female	5.6
491	Sibling	179		Female	5.7
492	Sibling	135		Male	5.1
493	T1D	157	55	Male	336.1
494	Sibling	116		Male	5.1.
495	Sibling	83		Female	4.9
496	T1D	117	5	Female	810.7
497	Sibling	69		Female	5.3
498	Sibling	22		Female	5.4
499	T1D	86	5 days	Female	706.3
500	Sibling	86		Male	5.1
501	T1D	100	4	Female	644.6
502	Sibling	56		Male	8.9
503	T1D	105	4	Female	148.4
504	Sibling	175		Male	8.4
505	Sibling	145		Female	8.7

GAD65 Ab ELISA	IA-2 Ab ELISA V2	ZnT8 Ab ELISA	IAA RIA	TPO Ab RIA
Units/mL	Units/mL	Units/mL	Units/mL	Units/mL
Cut off ≥5 u/ml	Cut off ≥7.5 u/ml	Cut off ≥15 u/ml	Cut off ≥0.4 u/ml	Cut off >0.3 u/ml
63.9	2754.0	11.0	0.7	nt*
0.9	1.1	8.0	0.0	0
0.0	1.3	7.8	48.1	0
0.6	9.2	7.6	0.0	0.1
120.5	435.4	7.7	48.2	0
0.0	0.0	8.0	0.0	0
>2000	30.7	7.8	0.5	nt
0.6	0.8	1.0	0.1	0
0.0	0.8	7.6	0.1	0
5.1	0.9	1.0	10.6	nt
0.0	0.0	0.0	0.1	0.1
0.0	0.0	7.9	0.1	0
0.0	0.8	7.6	0.1	0
37.1	0.8	7.7	41.8	>100
0.6	0.8	0.0	0.0	0
>2000	30.5	112.2	2.1	0.1
0.5	0.8	1.0	0.1	0.1
0.5	0.0	7.6	0.1	0
145.7	100.2	0.0	23.9	>100
0.6	0.8	4.3	0.1	0
>2000	0.9	1.0	17.8	nt
0.5	0.0	8.4	0.0	0
0.5	0.8	1.0	0.1	0
1768.4	0.0	641.4	12.9	nt
0.0	0.8	0.0	0.1	0
0.5	0.8	1.0	0.1	0
>2000	0.8	1.0	29.3	0.1
0.0	0.8	7.8	0.1	0.1
0.0	0.0	8.8	0.0	0
18.8	0.0	1.0	0.2	0
0.7	0.8	8.1	0.1	0
0.0	0.8	1.0	0.1	0
135.6	0.0	7.6	39.2	>100
611.6	0.0	1226.7	0.1	nt
0.6	0.0	4.5	0.0	0
>2000	1950.1	672.7	>50	nt
0.6	0.8	1.0	0.1	0
64.7	1.1	10.1	>50	0
67.9	0.9	7.7	0.1	0
0.5	0.0	7.6	0.0	0.1
1362.9	98.1	7.9	2.6	0
0.0	0.0	7.8	0.0	0
4.7	0.0	7.7	30.3	>100
>2000	0.0	7.6	0.0	0.1
0.5	0.0	8.0	0.0	0
14.4	0.8	8.9	>50	0
0.5	0.0	7.8	0.0	0.1
>2000	0.8	53.8	30.6	nt

0.0	0.0	9.3	0.1	0
0.0	0.0	11.2	0.0	0
41.0	142.7	108.5	8.2	0
0.6	0.0	7.8	0.0	>100
>2000	0.0	7.8	0.5	nt
1531.5	0.0	1.0	6.9	0.7
>2000	1.2	722.5	5.9	nt
1657.0	0.0	0.0	0.0	0
53.7	0.0	4.4	23.7	0
0.5	1.0	7.6	0.0	0
0.0	0.0	7.8	0.0	0
253.1	14.8	21.8	>50	2.8
0.6	0.0	1.0	0.1	0
856.0	0.0	7.7	>50	nt
0.0	0.0	0.0	0.0	0
0.6	46.0	1.0	>50	nt
0.5	0.0	7.6	0.0	0.1
0.5	0.0	0.0	0.1	0.1
0.6	0.0	7.8	0.0	0.1
28.1	23.5	8.4	37.9	0
0.5	0.0	4.3	0.0	0
0.6	19.4	14.2	6.0	37.6
0.0	0.9	8.1	0.0	0
175.2	12.0	9.0	>50	>100
786.8	13.0	7.9	0.2	0
7.8	0.9	164.3	20.5	0.1
0.5	5.9	7.7	0.1	0.1
0.5	0.0	7.7	0.0	0
1.0	4.3	7.5	>50	nt
0.5	0.9	7.5	0.1	0.1
1.0	0.9	7.5	0.7	0.1
0.0	0.9	7.6	0.1	0.1
0.5	1.0	7.8	0.1	0.1
109.1	1.1	7.5	24.5	>100
48.9	3.9	7.6	0.1	0.1
0.0	0.9	7.9	0.1	0
23.8	1.0	774.8	31.5	nt
55.3	54.2	9.4	5.1	nt
0.5	1.0	4.3	0.1	0.1
11.2	402.7	29.8	41.7	nt
0.0	1.4	89.1	0.1	0.1
0.7	1.9	4.2	23.4	nt
>2000	0.0	7.5	5.1	0
6.9	0.8	7.4	0.1	0.1
14.4	0.0	8.1	29.6	nt
0.6	0.9	8.4	0.1	0.1
168.8	1.1	4.2	37.3	0.1
0.5	0.8	8.8	0.1	0
540.2	0.8	7.6	>50	67.0
0.6	1.2	7.4	0.1	0
4.5	1.0	8.0	0.0	0
>2000	1.3	88.3	27.1	>100

0.0	0.7	4.0	0.0	0.1
0.0	0.0	4.5	0.0	43.8
>2000	29.8	490.0	>50	nt
0.0	0.0	3.9	0.1	0
0.0	0.7	4.4	0.1	0
0.0	0.0	6.5	0.0	0.1
1.0	0.0	1.0	0.0	0.1
43.4	347.8	19.0	0.8	1.8
0.6	0.0	2.6	0.3	0.1
1.3	0.9	20.2	0.9	0
0.0	0.0	4.3	0.0	0
0.0	0.0	0.0	0.0	0
>2000	2515.2	1183.5	13.3	nt
0.0	0.0	4.6	0.1	0.1
620.5	0.0	3.8	>50	>100
0.0	0.0	1.0	0.0	0
0.0	0.0	0.0	0.0	0
0.5	49.6	13.7	48.1	0
8.4	0.0	4.0	>50	0
0.0	1.2	3.8	0.0	0
0.0	0.0	4.2	0.0	0
217.1	0.0	4.0	>50	0
0.0	0.8	3.9	0.0	0
0.0	0.0	4.6	0.0	0
34.2	0.0	1.0	11.2	0
0.0	0.0	4.3	0.0	0
0.0	0.0	14.8	0.0	0
22.1	35.9	919.7	49.7	nt
0.0	0.0	3.9	14.8	0
192.0	0.0	6.1	6.3	>100
0.0	0.0	3.7	0.1	3.2
109.2	0.0	0.0	42.1	0
0.0	0.0	5.6	0.0	0
0.5	0.0	3.9	0.6	0.1
0.0	0.0	4.0	0.1	0
3.6	0.0	4.2	>50	>100
5.4	0.9	7.1	28.5	0
1.7	0.0	4.5	9.0	0.1
0.0	0.0	0.0	0.0	0.1
1518.3	0.8	6.5	28.2	>100
0.5	1.1	0.0	0.0	0
0.6	0.0	0.0	0.0	0
240.5	0.8	0.0	7.1	22.7
1575.5	0.8	1.0	0.0	0
55.0	1.4	4.5	27.2	0.4
0.0	0.0	4.5	0.0	0
38.0	0.0	62.3	2.6	0.2
0.5	0.0	1.0	0.0	0.1
6.3	0.8	4.5	22.7	0.1
0.0	0.8	5.3	0.0	0
0.0	0.0	1.0	0.0	0.1
0.5	0.0	4.4	0.0	0

0.0	0.0	0.0	0.0	0
0.6	0.0	4.5	0.0	0.1
0.0	0.0	4.4	0.0	nt
0.0	0.0	0.0	0.0	0.1
22.6	0.8	4.7	43.9	nt
0.5	0.0	0.0	0.0	0
0.6	0.0	5.4	0.0	0
52.3	126.8	301.6	>50	0.1
0.5	0.0	0.0	0.1	0.1
>2000	29.7	306.6	>50	>100
45.8	0.8	0.0	**0.38**	0.1
0.0	0.0	4.5	0.1	0.1
118.1	61.6	181.9	>50	0
0.0	2.7	6.9	0.1	nt
0.0	0.0	4.5	0.0	0
240.6	0.0	60.4	25.8	nt
0.0	0.0	1.0	0.0	0
630.3	1760.1	528.5	39.5	nt
0.7	0.8	0.0	0.1	0.1
259.2	0.9	526.6	17.4	nt
0.5	0.0	4.5	0.0	0
0.7	0.9	3.9	0.1	0.1
0.5	0.0	4.3	20.9	0
0.0	0.0	1.0	0.1	0.8
3.6	62.1	2.5	10.4	0
0.7	0.8	4.4	0.0	0
25.4	2.5	4.6	10.3	>100
0.7	0.8	4.1	0.0	0.1
0.7	0.8	1.0	0.0	0.1
0.7	2.1	4.3	0.1	3.5
>2000	3.8	4.7	30.1	0.1
>2000	0.0	0.0	0.1	0
24.2	0.8	197.1	>50	nt
0.6	0.8	0.0	0.0	0
7.5	22.1	1073.7	4.2	nt
0.6	0.0	4.8	0.0	0
0.7	0.8	4.0	0.0	0
47.5	297.3	685.3	1.6	nt
>2000	71.6	22.4	22.6	0
11.9	719.6	5.3	0.1	0
0.8	0.0	4.5	0.1	0
0.7	0.0	4.1	0.0	0
>2000	0.0	224.7	0.0	>100
2.5	0	5.6	0.1	0
>2000	30.9	6.6	31.1	>100
>2000	0	8.6	>50	9.2
0	0	8.5	0	0
>2000	1.0	529.1	0.1	0
0.7	0	5.5	0	0
>2000	367.5	256.4	4.9	0
1.0	0	5.7	0.1	0.1
12.0	0	5.2	28.9	>100

0.7	0	6.7	0.1	0
186.7	1.8	37.3	>50	0
12.7	0	4.7	39.4	0
0.5	19.7	4.5	0	0.1
0.9	0	4.8	40.3	0.1
0.9	10.9	4.6	0.1	0.1
0.7	0.9	3.6	0.1	0
6.0	0	4.5	10.8	0.1
50.1	9.1	5.4	0.3	nt
958.9	5.0	5.0	35.2	>100
0.5	0	4.8	0	0
5.1	0	52.9	0.1	0
0.7	0	4.8	0.1	0.1
71.0	0	6.4	8.0	0
1.2	0.8	6.9	0	0
6.5	0	6.9	2.3	nt
1.2	0	7.7	0	7.7
>2000	0	5.9	>50	nt
0.5	0	4.5	0	0
145.9	0	89.9	24.0	0
0.7	0	7.7	0	0
1.3	0	5.5	0.1	0
254.9	18.8	90.4	0.3	nt
1.0	0	7.3	0	0
39.5	0	13.2	14.0	nt
3.3	0	6.0	>50	>100
1.3	0	5.7	0	0
1034.3	0	4.1	0.7	76.3
0.6	0.9	7.9	0.1	nt
0.6	0	3.6	0	0
>2000	2.5	4.5	1.4	nt
0.5	0.8	4.6	0	0
0.6	0.9	4.8	0.1	nt
0.8	4.1	9.7	0.2	0.1
38.0	157.1	77.7	0.1	17.9
0.9	0.8	5.4	0	0
59.0	1.8	8.3	0	1.9
>2000	675.0	169.4	0.6	nt
2.5	0.9	4.4	0.1	>100
5.5	1.3	494.1	0.1	0
0.5	0.7	6.8	1.1	nt
463.6	439.7	226.1	11.7	0
0.8	1.0	5.6	0	0.1
0.6	0	5.0	0	0
767.2	56.3	5.0	11.9	4.5
0.6	0	5.7	0	29.5
0.7	0	5.8	0	0
1.9	0	27.7	0.1	0
0.5	0	4.6	0.1	0
0.8	13.5	6.0	0	nt
0.5	0.7	4.8	0.1	0.1
0	0	6.4	0	0

>2000	2770.7	930.5	1.1	0
0.6	0.8	6.1	1.7	0.1
10.1	0	9.9	0	0
>2000	0	3.7	38.8	13.2
0.6	0	0	0	0.1
43.5	0	379.6	46.3	0
30.9	37.9	1434.6	1.0	0.1
0.6	1.1	18.8	12.3	nt
7.2	78.2	13.2	2.4	nt
55.1	0	15.2	0.7	1.2
34.5	0.9	4.8	0	0
0.6	1.3	5.0	0.3	0.1
89.0	0	6.1	13.1	0.28
0	0	4.3	0	0.1
514.7	0	8.2	18.1	0
1.3	12.3	4.7	0.1	nt
1.5	0.7	4.6	0	0.1
24.8	0	12.0	1.1	0
1.1	0	4.5	0	0
0	0	3.7	0.1	0
0	0	3.6	0.1	nt
0	0	5.1	0	0.1
>2000	0	4.3	2.5	0.1
0.9	0	3.9	0	0
0	0	6.9	13.2	nt
1.4	1.6	4.0	0.1	0.1
2.7	0	4.2	3.9	nt
1.4	0	3.6	0.1	0
1.2	0	4.8	0.1	0
0	0	4.1	0	0
0	0	4.4	0	0.1
1.6	0	8.2	>50	>100
0.5	0	3.7	0.1	0.1
11.1	715.9	539.6	1.7	0
1.4	0	4.2	0	0
>2000	0	1017.1	2.8	nt
1.1	0	2.3	0	0
0	0	2.3	0.1	0
2.7	0.7	4.7	0.0	0.1
8.9	0.9	6.1	15.6	0
0.0	0.0	4.4	0.1	0.1
2.0	0.8	4.5	15.0	>100
0.0	0.8	4.4	0.0	0.1
>2000	2.5	133.1	45.1	0.1
0.0	0.0	2.7	0.0	0.1
11.0	1.0	1.0	16.8	0
0.0	0.9	4.7	0.0	0
331.9	2.7	842.9	44.5	0
0.0	0.7	4.9	0.0	0.1
0.0	21.0	80.7	>50	0
194.9	0.8	43.7	32.4	14.8
1.1	0.0	0.0	0.1	0.1

>2000	0.0	1.0	12.5	0
131.4	464.2	259.8	6.9	0
0.0	0.0	2.7	0.0	0.1
1010.8	0.0	4.3	7.7	1.6
1.6	0.0	1.0	0.0	0
0.0	1.0	1.0	0.0	0
9.1	218.2	263.6	46.2	0.1
10.4	1.3	6.7	0.0	0.1
291.5	14.6	11.0	0.1	nt
238.7	0.0	7.4	2.8	0
1.6	0.0	2.6	0.0	0.1
1.8	464.4	640.5	30.1	0
1541.4	1.1	98.4	14.4	0
>2000	394.7	657.0	15.3	0
6.7	0.0	2.6	0.0	0
31.5	0.0	89.8	0.3	0.1
>2000	0.0	8.7	10.8	>100
1.4	0.0	4.5	0.0	0
1.0	0.0	4.3	0.1	3.0
60.0	0.8	53.3	6.3	0.1
1.5	0.0	0.0	0.0	0
0.0	0.8	4.3	0.0	0.1
0.5	0.0	4.2	0.0	0.1
>2000	19.9	406.6	22.5	>100
2.3	1.0	4.9	0.1	0
>2000	0.7	4.8	0.1	0
0.0	0.8	4.1	0.0	0.1
>2000	0.8	6.5	>50	nt
0.5	0.8	4.6	0.0	0
0.0	0.7	1.0	0.1	0
1.5	5.0	6.2	22.5	0.1
0.5	0.7	4.8	0.2	0
1.3	0.7	4.6	0.1	0
0.0	0.0	1.0	0.1	0
81.6	0.0	83.3	20.5	0
>2000	339.0	803.9	26.5	>100
1.2	0.0	5.8	0.1	0
34.6	0.9	7.2	>50	0
0.0	0.7	0.0	0.0	0
293.2	12.9	5.1	2.2	>100
1.3	0.0	4.6	0.1	0
9.8	0.0	4.4	1.5	0
1.0	0.7	4.8	0.0	0
1.0	0.7	8.2	0.0	0
0.5	0.7	4.4	0.0	0
76.8	22.9	72.1	12.5	0
1.2	0.0	4.4	0.1	0
19.1	0.0	173.4	13.8	0
1.3	1.1	4.9	0.0	0
1.2	1.4	4.9	0.2	0
125.7	0.7	4.3	0.0	0
0.0	0.0	4.6	0.0	0

1.2	0.0	4.6	0.0	nt
0.0	0.7	2.6	0.1	29.6
0.5	0.0	4.2	0.1	0.1
9.4	0.9	0.0	2.9	1.5
0.0	0.8	0.0	0.0	0
468.8	25.3	601.7	0.9	0
0.0	1.2	0.0	0.0	0
3.6	1.0	3.7	11.4	0
0.0	1.1	0.0	0.0	0
1.4	1.4	3.9	24.7	0.1
0.0	1.1	0.0	0.0	0
3.2	392.9	648.3	10.5	0
>2000	151.5	231.2	17.7	4.2
8.2	0.0	0.0	0.1	0
0.0	1.0	0.0	0.1	0.1
1.3	0.8	0.0	0.1	0.1
0.0	0.7	0.0	0.1	0.1
879.2	0.9	0.0	10.7	0.1
0.0	1.0	0.0	0.1	0.1
0.0	0.9	0.0	0.1	0.1
>2000	1.0	522.4	20.5	64.3
0.0	1.7	0.0	0.1	0.1
0.0	0.9	0.0	0.0	0
1.9	0.0	0.0	0.1	0
54.0	1.1	0.0	2.8	0
1.2	0.9	0.0	0.0	0.1
1242.2	0.9	116.8	>50	23.4
0.0	0.8	0.0	0.1	nt
>2000	0.8	103.2	0.1	0
229.3	1.1	5.7	24.6	nt
0.8	2.7	2.6	0	0
0.6	0.9	25.3	0.1	0
6.2	76.0	181.0	>50	0.1
1721.4	0.9	59.4	14.3	nt
0.7	0	2.2	0.1	0
2.8	211.4	624.0	0.1	0
0.8	0.8	0	0	0
8.7	0.8	0	>50	0.1
0.8	1.0	0	0	>100
0.6	0.7	0	0.1	0.1
1.4	0	3.2	21.9	0
3.6	1.1	130.2	13.2	0
0.9	0	2.2	0.1	0.1
1.8	0.8	0	0.1	0.1
0.7	0	0	0	0
>2000	0.8	0	46.3	11.7
0.6	0.7	2.5	0	0
>2000	0.8	501.0	41.7	nt
11.3	0	0	0	0
0.8	1.4	23.3	0.2	0.1
0.7	0	0	0.1	0.1
191.7	1500.0	646.0	10.2	nt

23.0	0	0	6.1	0.1
0.7	0	4.5	0.1	0
>2000	0.7	247.7	16.2	3.2
0.6	0	0	0.1	0.1
111.2	24.2	270.1	35.7	>100
0.6	0	4.1	0	0.1
8.3	192.4	2.5	36.1	0
0.7	1.8	2.0	1.1	0
97.7	1.0	0	1.9	0.1
0.5	0	0	0	0.1
0.8	0.8	0	0.2	0.1
974.0	12.8	464.6	20.8	0.1
0.6	0	0	0.1	0
0	0.8	0	0.1	0.1
0	0	5.7	0.4	0.1
94.0	0.9	378.3	35.3	0
210.4	1.1	0	22.8	0.1
3.1	35.5	1.0	46.7	nt
0	0	2.0	0.3	0
0.5	0	12.4	0.1	0.1
0	0.8	0	0.1	0.1
460.6	0	180.3	0.1	0.1
501.7	384.5	42.1	5.1	0.1
0	0	1.8	0	0.1
16.2	596.3	8.8	17.9	0
0	0	3.8	0	0
0.5	0	1.0	0.9	0.1
>2000	0.8	19.5	4.3	0.1
0	0.9	0	0	0.1
>2000	600.1	0	0.8	0.1
13.4	25.6	107.5	29.9	>100
2.0	1.8	0.0	0.1	0
1.0	1.0	1.0	0.3	0
0.5	0.8	1.0	0.1	0.1
68.7	0.8	133.0	22.4	0.1
42.6	35.2	14.7	0.7	nt
1.2	0.8	1.0	0.2	24.5
11.0	652.6	974.7	2.6	0
>2000	0.9	2.2	34.9	0.1
0	1.1	1.5	0.4	0
>2000	0.8	58.9	>50	0
0	0.9	0	0.7	0
0.5	1.1	1.8	0.1	0
0	0.9	1.0	0.1	0
0.5	1.0	1.8	0.5	0
0.5	1.2	2.4	0.9	0
0.8	1.4	4.2	0.8	nt
>2000	0.7	586.0	6.0	0
0	0	0	0	0
2.4	0.0	0	0	0
0	0.0	0	0	0
34.2	1.1	40.6	>50	0

0	0.0	0.0	0	0
>2000	31.3	511.0	1.3	0
0	0.8	2.8	0	0.1
0	1.0	0	0	0
0	1.0	3.8	0	0
2.2	1.0	0	0.2	0
0.8	0.9	0	0	0.1
0.5	0.8	0	0	0
0	0.9	0	0.2	0
0	0.8	0	0	0
99.9	37.2	2.2	20.2	0
0	0	3.8	0	0
0.9	0.7	0	0	0
482.3	784.9	315.7	9.2	12.7
0.5	0.8	0	0	0
0	0.8	0	0	nt
31.2	164.9	812.8	0.1	0.1
0	0.8	0	0.1	0.1
8.6	182.7	378.1	2.0	0.1
0.6	1.9	0	0	0
17.5	36.8	4.1	0.1	0
0.6	0.9	0	0	0
1.4	1.1	0	0	0

Tg Ab RIA Units/mL	ELISA RSR TRAb 3rd Generation Units/L
Cut off >0.3 u/ml	Cut off ≥0.4 u/L
nt	nt
0	0
0.1	0.1
0.2	0.1
0.2	-
0.1	0
nt	nt
0.2	0
0.2	0.1
nt	nt
0.1	0.1
0	nt
0.1	0
75.5	nt
0.1	nt
0.2	nt
0.2	nt
0.2	0
>100	0
0.1	nt
nt	nt
0.1	0.1
0.6	nt
nt	nt
0.2	0.1
0.1	nt
0.2	0.1
0.1	0
1.2	0
0	0.1
0.1	0.1
0.2	0.1
>100	0.1
nt	nt
0.2	0.1
nt	nt
11.7	0.1
0.2	0.1
0.2	0.1
0.2	nt
0.2	0.1
0.2	0.1
45.2	0.1
0.9	0.1
0.5	0.1
0.8	0.1
0.7	0.1
nt	nt

0	0.2
0	0.1
0	0.1
24.7	0.1
nt	nt
0.1	0.1
nt	nt
0.2	nt
0	nt
0.1	0
0.1	nt
0.2	-
0.1	0.1
nt	nt
0	0.1
nt	nt
0.1	0.1
0.1	nt
0.1	0.1
0	nt
0	0.1
13.9	0.1
0.6	0.1
52.7	nt
0.6	nt
0.2	0.1
0.2	0.1
0	0
nt	nt
3.6	0.1
0.2	0.1
0	0.1
0.1	0.1
2.6	3.3
0.2	0.1
0.1	0.1
nt	nt
nt	nt
0.4	0.1
nt	0.1
0.1	0.1
nt	nt
0.1	nt
0	0
nt	nt
0.1	0
0.1	0.2
0.1	0
3.9	0.1
20.6	nt
0.2	0
95.8	0.1

5.2	0.1
25.9	0
nt	nt
0.1	nt
0.1	0
0.1	0
0.2	0
0.2	0.1
0.1	0
0	0.1
0.1	0
0	0
nt	nt
0.2	0
63.8	0.2
0	0.1
0	0
0	0.2
0.2	0
0	0
0	0.1
0	0.1
0	0
0.1	0.1
0.2	0.1
0.1	0
0	0.1
nt	nt
0.1	0
26.3	0
57.2	0
0	0.1
0.6	0.1
0.1	0.1
0.2	0.1
>100	nt
0.1	0.1
0	0.1
0	0
81.0	0.2
0.1	0
7.2	0.1
23.8	nt
0.1	0.1
10.8	nt
0.2	nt
3.4	0.1
0.2	nt
0.2	0
0.2	0
0.2	0
0.5	0.1

0.2	0
0.2	0
nt	nt
0.2	0
nt	nt
0.2	0
0.1	0
0.1	0.1
0.2	0.1
>100	0.1
0.4	0.1
0.5	0
0.1	0
nt	nt
0	0
nt	nt
0.4	nt
nt	nt
0.2	0
nt	nt
0	0
0.1	0.1
0.1	nt
0.31	nt
0.1	0
0.1	nt
14.4	nt
0.5	0.1
7.2	0.2
12.8	0.1
9.2	0
0	0.1
nt	nt
0	0.1
nt	nt
0	0.1
0.1	nt
nt	nt
0.1	nt
0.1	nt
0.1	nt
0	0
20.1	nt
0	nt
>100	nt
14.3	nt
0	0.1
0.2	0
0	0
0.1	nt
0.1	nt
8.0	0.1

0.2	0
0.1	nt
0.1	0.1
0.1	nt
0.1	nt
0.1	0.1
0.1	nt
0.1	0
nt	nt
28.9	0
0.2	0
0.1	0
0.1	0.1
0.1	nt
0	nt
nt	nt
17.2	0
nt	nt
0.1	0.1
0	0.1
0	0.1
0.2	0.1
nt	nt
0	0.1
nt	nt
36.8	nt
0.1	nt
1.0	0
nt	nt
0	0
nt	nt
0	0.1
nt	nt
0.2	nt
23.9	0
0.1	0
0.26	0.1
nt	nt
42.0	0
0.1	nt
nt	nt
0.1	0.1
0.1	0
0.1	0
24.0	0
78.8	0.1
0.1	0.1
0.1	0.1
0.1	0
nt	nt
0.2	0.1
0.1	0.1

0	0
0.34	0.1
0.1	0.1
1.2	0.1
0.2	0
0.2	0.1
>100	0.1
nt	nt
nt	nt
15.8	nt
0	0.1
0.1	nt
37.3	nt
0.2	0.1
0.1	0.1
nt	nt
0.1	0
0	0
0.1	0.1
0.26	0.1
nt	nt
0.1	0.1
0.1	0.1
0.1	0
nt	nt
0.1	0
nt	nt
20.8	0.1
0.2	0.1
0	0.1
2.0	nt
3.2	0.1
31.1	0.1
0.1	0.1
0	0.1
nt	nt
0.1	0.1
0	0.1
0.1	0.1
0	0
0.2	0.1
43.4	0.1
0.2	nt
0.1	0.1
1.4	0.1
0	0.1
0.1	0.1
0	0
0.2	0
0	0
25.3	0.1
0.1	0.1

0	0.1
0	0
0.2	0.1
0	0.1
0	0.1
0	nt
0.1	0
0.1	0.1
nt	nt
0.1	0.1
0	-
0	0.1
0	0.1
0	0.1
0.1	0.1
0.2	0.1
>100	0.1
0.1	0.1
0.2	0.1
0	0.1
0.2	0.1
0.4	0.1
0.2	0.1
>100	0.1
0.1	0.1
0.5	0.1
0.2	0.1
nt	nt
0.1	0.1
0.26	0.1
0.2	0.1
0.1	0.1
0.2	0.1
0.1	0.1
0	0.1
21.8	0.1
0.4	0.1
0.1	0.1
0.1	0.1
11.6	nt
0.1	0.1
0.1	0.1
0.1	0.1
0.1	0.1
0.1	0.1
0.1	0
0.2	nt
0.1	0
0.1	0.1
0.1	nt
0.1	0
52.5	0.1

nt	nt
21.9	0.1
8.4	0.1
0.2	nt
0.7	0
0.1	nt
0	0
0.2	0
0.1	nt
0.1	0
0.1	0
0.1	nt
0.1	nt
0.1	0.1
0.3	0
0.32	0.1
0.1	0.1
0.1	0.1
0.1	0
0.26	nt
48.4	0.1
0.2	nt
0	0
0	0.1
0.1	0
0.1	nt
35.1	0.1
nt	nt
0	0
nt	nt
0	0.1
0.2	0.1
0.6	0
nt	nt
0	nt
0.1	0.1
0.1	0.1
0.2	0
56.2	0.1
0.1	nt
0	nt
0.1	nt
0.1	0.1
0.1	0.1
0.1	0.1
0.1	0.2
0	0.2
nt	nt
0.1	0.1
0.1	0
0.1	nt
nt	nt

0.1	0.1
0.1	nt
13.0	0.1
0.1	nt
50.5	0
0	0.1
0.1	nt
0	0
0.2	0.1
0.1	0.1
0.2	0.1
0.1	nt
0.1	nt
0	0.1
0.1	0.1
0.1	0.1
0.1	0.1
nt	nt
0	0.1
0.2	nt
0.1	0
0.2	0.1
0.1	nt
0.1	0.1
0	0
0.1	0.1
0	1.2
3.0	0.1
0	0.1
0.2	0.1
11.3	nt
0.8	nt
0	0.1
0	0.2
0.26	0
nt	nt
26.7	nt
0.1	nt
0.2	0
0	0.1
0.1	0
0.1	0
0.2	0
0	0
0	0
0.1	0
nt	nt
0.1	0
0.1	0
0	0.1
0	0.1
0	0.1

0	0.1
0	0
29.1	0
0	nt
0.1	0
0	0
0.1	0
0.1	nt
0.2	0
0	0
0	0.1
0.1	0.1
0	0.1
24.1	0.1
0.1	nt
nt	nt
0.1	0.1
0.1	nt
0.6	0
0.1	0
0.1	0
0.1	0
0.1	0

nt* = not tested
