# Supplementary figures and images for: The prevalence of diabetes and thyroid related autoantibodies in Sri Lankan children with type 1 diabetes and their unaffected siblings – The utility of a new screening assay
Source: Front Endocrinol (Lausanne). 2023 Feb 6;14:1028285. doi: 10.3389/fendo.2023.1028285 (PMC9939822; doi:10.3389/fendo.2023.1028285)

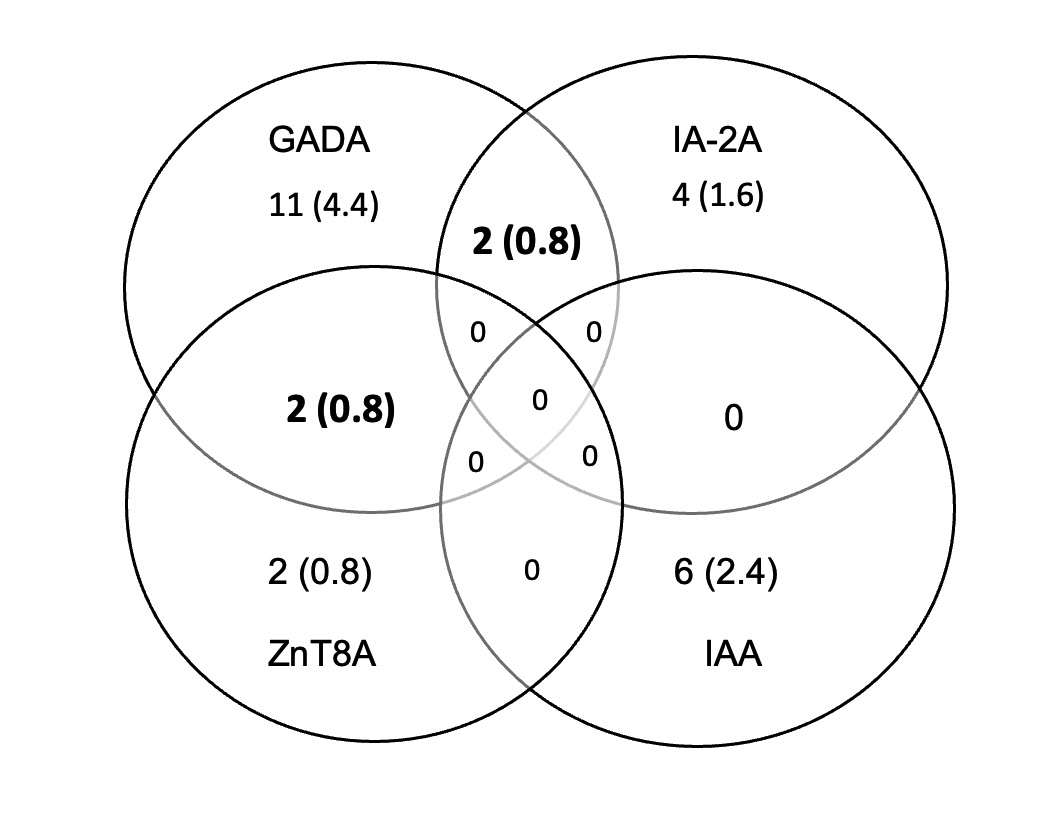

Supplement: Supplementary file 2 [file Image_1.jpeg]
